# HIV-1 Env C2-V4 Diversification in a Slow-Progressor Infant Reveals a Flat but Rugged Fitness Landscape

**DOI:** 10.1371/journal.pone.0063094

**Published:** 2013-04-29

**Authors:** S. Abigail Smith, Charles Wood, John T. West

**Affiliations:** 1 Department of Microbiology and Immunology, University of Oklahoma Health Sciences Center, Oklahoma City, Oklahoma, United States of America; 2 Nebraska Center for Virology, University of Nebraska, Lincoln, Nebraska, United States of America; 3 School of Biological Sciences, University of Nebraska, Lincoln, Nebraska, United States of America; INSERM, France

## Abstract

Human immunodeficiency virus type-1 (HIV-1) fitness has been associated with virus entry, a process mediated by the envelope glycoprotein (Env). We previously described Env genetic diversification in a Zambian, subtype C infected, slow-progressor child (1157i) in parallel with an evolving neutralizing antibody response. Because of the role the Variable-3 loop (V3) plays in transmission, cell tropism, neutralization sensitivity, and fitness, longitudinally isolated 1157i C2-V4 alleles were cloned into HIV-1_NL4-3_-eGFP and -DsRed2 infectious molecular clones. The fluorescent reporters allowed for dual-infection competitions between all patient-derived C2-V4 chimeras to quantify the effect of V3 diversification and selection on fitness. ‘Winners’ and ‘losers’ were readily discriminated among the C2-V4 alleles. Exceptional sensitivity for detection of subtle fitness differences was revealed through analysis of two alleles differing in a single synonymous amino acid. However, when the outcomes of N = 33 competitions were averaged for each chimera, the aggregate analysis showed that despite increasing diversification and divergence with time, natural selection of C2-V4 sequences in this individual did not appear to be producing a ‘survival of the fittest’ evolutionary pattern. Rather, we detected a relatively flat fitness landscape consistent with mutational robustness. Fitness outcomes were then correlated with individual components of the entry process. Env incorporation into particles correlated best with fitness, suggesting a role for Env avidity, as opposed to receptor/coreceptor affinity, in defining fitness. Nevertheless, biochemical analyses did not identify any step in HIV-1 entry as a dominant determinant of fitness. Our results lead us to conclude that multiple aspects of entry contribute to maintaining adequate HIV-1 fitness, and there is no surrogate analysis for determining fitness. The capacity for subtle polymorphisms in Env to nevertheless significantly impact viral fitness suggests fitness is best defined by head-to-head competition.

## Introduction

Human immunodeficiency virus type I (HIV-1) is a positive-sense RNA virus that replicates via error-prone reverse transcription and undergoes inter-strand recombination, introducing approximately 3.4×10^−5^ mutations/base pair per replication cycle [1], [2]. These diversification mechanisms result in HIV-1 populations behaving as large, dynamic groups of related, yet genetically distinct, organisms whose evolutionary characteristics can be modeled as a quasispecies [3]. Quasispecies diversity provides HIV-1 with evolutionary flexibility to respond to environmental selective pressures while maintaining capacity to produce replication-competent progeny.

HIV-1 diversity facilitates evolution of resistance to antiretroviral therapy and escape from host immune responses [4]–[15]. Effective antiretroviral drugs, such as nucleoside and non-nucleoside reverse transcriptase inhibitors, protease inhibitors, and integrase inhibitors, have been developed to impede enzymatic activities required for HIV-1 replication. [16]. However, due to HIV-1 quasispecies diversity, drug resistance develops within many patients, and transmission of drug resistant variants between patients remains a concern. The CD8^+^ T-cell recognition of MHC class I restricted HIV-1 epitopes presented by infected cells is thought to play a critical role in controlling viral load [17]–[21]. While some MHC I alleles have been associated with decreased viral load and improved patient prognosis, intrapatient quasispecies diversity also supports escape from cytotoxic T lymphocyte (CTL) responses in most individuals [22], [23]. More disturbingly, there are indications that the global HIV-1 population may be adapting to avoid CTL selective pressure [24]–[26]. Therefore, large dynamic populations of subtle sequence variants allow HIV-1 continue to replicate despite potent environmental selection imposed by treatment and the immune system.

For our study, as well as many described below, fitness is defined as the replicative capacity of a viral variant in a defined environment [27]. Using this definition, a ‘survival of the fittest’ landscape, in its simplest form, can be modeled on a two-dimensional fitness coordinate system as a series of discrete peaks representing highly fit variants, and valleys representing low fitness variants. HIV-1 sequence diversity in Gag, Nef, Pro, and RT allows viral escape from selective pressures. However, escape mutants often have less replicative capacity; they are less ‘fit’ *in vitro* than the parental strain [6], [8], [25], [28], in the absence of the selecting agent. Reductions in *in vivo* fitness, can lead to decreased viral load, which in turn, hinders disease progression and potential for virus transmission [29]–[33].

Humoral immunity, typified by neutralizing antibody (NAb) responses against HIV-1 Env, develops within months of infection in most patients [34]–[37]. The host NAb response exerts selective pressure on Env leading to the presence of hypervariable regions (V1–V5) that are thought to undergo almost continual variation and selection. The variable regions alternate with relatively conserved regions of Env (C1–C5) that are generally considered to be immunologically ‘silent’ or highly functionally constrained [38]. Diversification and selection allow Env to escape neutralization over the course of infection [34], [37], [39], [40]. Based upon the observations that CTL and antiretroviral escape mutations in Gag, Nef, Pro, or RT, incur a fitness cost, one might anticipate that a similar fitness penalty would be evinced by Env escape mutants. However, despite the fact that a majority of infected individuals mount a neutralizing response against Env, clearance of HIV-1 has never been demonstrated, implying that escape from NAb does not come at a profound fitness cost. This suggests that Env evolves to maintain fitness while exploring diverse sequence space, a concept termed mutational robustness, or ‘survival of the flattest’ [41], [42]. A two dimensional representation of this concept would be a fitness plateau where many variants in sequence space are compatible with adequate replicative fitness.

In the absence of CTL, NAb, or pharmacologic selection, HIV-1 replicative fitness has been correlated with early events in the replication cycle including receptor binding, fusion, and entry; all steps mediated by Env [43], [44]. In investigations of relationships between HIV-1 fitness and disease progression, Quiñones-Mateu *et al*., found that fitness, assayed *in vitro,* correlated with disease progression *in vivo* [45]. More importantly, *env* recombinant viruses duplicated the fitness stratification, suggesting that HIV-1 fitness, in the absence of drug selection, was a function of Env-mediated processes. Subsequent reports verified these observations with alternative approaches and patient samples [43], [44], [46], [47]. Troyer et al. investigated temporal relationships in the V3-loop region of HIV-1 in subtype B infected adults and reported a modest correlation between Env diversification and fitness, as well as the anticipated higher fitness of X4- versus R5-tropic strains in individuals who underwent a coreceptor switch. However, the longitudinal effects of Env diversification on viral fitness in infected children and particularly those with HIV-1 subtype C, where R5-X4 transition is less common, has not been longitudinally investigated. Moreover, the molecular or functional determinants of fitness within Env remain to be fully elucidated.

Large differences in HIV-1 fitness can be identified via viral growth kinetics, either by comparatively assaying rates of increase in reverse transcriptase activity or accumulation of retroviral proteins; however, these approaches cannot elucidate subtle fitness differences between HIV-1 variants [15]. In the absence of gross replicative defects, two variants can have virtually identical *in vitro* growth kinetics, yet disparate fitness values [45], [46], [48]. Thus, viral fitness is optimally defined by experimentally quantifying viruses in dual-infection competitions [49]. Recently, recombination between a reporter provirus and Env PCR products has been used to produce infectious HIV-1 containing a fluorescent reporter gene. Competitions between one virus with an eGFP reporter and another containing a DsRed2 reporter allowed for quantification of HIV-1 fitness values by flow cytometry [50].

To support the more specific molecular cloning of various *env* domains, we modified these fluorescent reporter infectious molecular clones by introducing a series of silent restriction sites into *env*. These unique constructs support precise creation of envelope chimeras, allowing for localization of Env-mediated phenotypes to specific regions (ex. ectodomain), specific domains (ex. V1–V5 loops) or precisely defined domains (ex. V3-loop). The C2-V4 region contains the Env third variable loop (V3), a major determinant of cell tropism, coreceptor usage, and transmission [51]–[55]. Based on its important role in Env function, it is not surprising that V3-loop polymorphisms have been associated with changes in viral fitness, that antibodies targeting V3 can neutralize viral infectivity, and that the V3-loop determines susceptibility to entry inhibitors [28], [56]–[59]. Previously, we showed that the V3-loop sequences from a treatment naïve, subtype C-infected, slow-progressor, Zambian infant diverged extensively from birth to 67 months of age. Using viral isolates derived by co-culture, divergence was correlated with an evolving Ab response that, at each time-point, neutralized previous isolates but lacked efficacy against the contemporaneous viral isolate [60].

Given the lack of a coreceptor switch or disease progression in the infected child, we sought to define relationships between Env C2–V4 sequence divergence, glycoprotein function, and viral fitness. Patient sequences derived over a 67-month sampling regimen were introduced into each of the HIV-1_NL4-3_ eGFP or DsRed2 infectious molecular clones. The resulting virus preparations were subjected to competitive dual-infection matrices to determine viral fitness of each C2-V4 allele relative to all others from the same patient. To test for correlation of Env functional parameters with fitness, the Env chimeras were then subjected to biochemical characterization for Env synthesis and processing, incorporation into virions, affinity for CD4 and CCR5, and RT-normalized infectivity.

Here we demonstrate the utility of a modified system for quantifying Env-mediated fitness, or replication capacity, where patient-derived Env sequences can be directly cloned into infectious molecular clone reporter viral constructs. In experiments using subtype C Env C2-V4 sequences from an slow-progressor child, we show the capacity to detect ‘winners’, ‘losers’, and ‘ties’ in individual competitions, including detection of fitness differences between extremely subtle polymorphisms. Aggregate analysis of fitness/replication capacity associated with polymorphisms in the 1157i C2-V4 region of Env, supports a flat, but rugged, fitness distribution indicative of mutational robustness.

## Results

### Construction and Characterization of HIV-1 NL4-3 MSS-eGFP and -DsRed2 Infectious Molecular Clones

HIV-1 fitness has been studied previously using fluorescent reporter HIV-1 vectors lacking either *env* or *pol* sequences that, when linearized and co-transfected with patient-derived PCR products, recombine to generate infectious chimeric virions. This system offered substantial improvement over heteroduplex-based fitness assessment regimens by supporting quantification of outcomes at the single cell level using flow cytometry [45], [61]. However, for our purposes, the method still suffered from limitations. First, the production of viable progeny virions results from inherently inefficient processes, co-transfection of a linearized vector and a PCR product, followed by their subsequent recombination. Second, the system only allowed for the insertion of pre-defined patient *env* sequences since recombination requires sequence homology between the linearized *env*-deleted vector and the PCR product to be inserted. Moreover, almost any instance of illegitimate recombination is likely to produce stop codons and thereby non-infectious progeny, and, as we will show, even subtle variations in amino acid sequence can have significant effects on Env mediated fitness. Therefore, it was important to be able to define the junctions of the inserted sequence and the vector with certainty. We further reasoned that it would be useful to have the flexibility to concentrate on specific functional domains of *env*, or to pursue a reductionist approach in analyses from a complete Env sequence down to domains associated with particular phenotypes.

**Figure 1:**
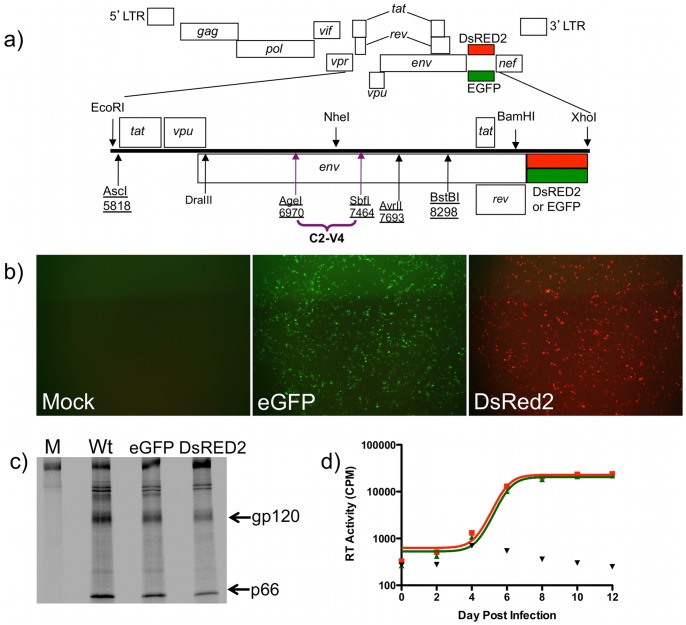
Expression of infectious molecular clones results in fluorescence in 293T transfectants and generation of infectious virions. **a)** Diagram of the HIV-1 NL4-3-MSS-eGFP/DsRED2 construct showing an expansion of the region bounded by EcoRI and XhoI and encompassing the code for Tat, Vpu, Rev, and Env. Introduced silent mutations are underlined. Sequence region utilized in this paper purple. **b)** HEK 293 cells transfected with either NL4-3-MSS-eGFP or NL4-3-MSS-DsRED2 were imaged by fluorescent microscopy (40×) at 48-hours post-transfection (b). **c)** Pelleted virions derived from ^35^S- radiolabeled transfected cells were immunoprecipitated with HIV-Ig and the resulting proteins were separated by SDS-PAGE revealing bands corresponding to gp120 (Env) and p66 (RT) in wild-type, NL4-3-MSS-eGFP, and NL4-3-MSS-DsRED2, but not mock transfected cells. **d)** Virus stocks derived from NL4-3-MSS-eGFP (green line) and NL4-3-MSS-DsRED2 (red line) transfections were used to infect U87.CD4.CXCR4 cells. Virus replication kinetics were assayed using a ^3^H-based RT assay at the indicated time-points. Mock transfected cell supernatant served as a control (black triangles).

We used site-directed mutagenesis to introduce numerous silent restriction sites into the HIV-1_NL4-3_ *env* gene. The result was pNL4-3-MSS (Multi-Silent Site), which improved the flexibility and efficiency of making Env-chimeras by creating a mechanism to clone virtually any Env sequence (**Fig. 1A**). To capitalize on the exceptional utility of the partial genome fluorescent reporter vectors produced by Weber *et al.* [50], we transferred the fluorescent reporter sequences from pNL4-3-Δenv-eGFP and pNL4-3-Δenv-DsRed2 into pNL4-3-MSS to create pNL4-3-MSS-eGFP and pNL4-3-MSS-DsRed2. Both infectious molecular clone fluorescent constructs were sequenced to ensure there were no unanticipated mutations (data not shown). Cells transfected with pNL4-3-MSS-eGFP and pNL4-3-MSS-DsRed2 express the respective fluorescent proteins from the HIV-1 LTR promoter (**Fig. 1B**), providing a convenient evaluation of transfection efficiency and a surrogate, *in situ* marker for HIV-1 gene expression. To determine whether the constructs produced virus particles, transfected cells were metabolically labeled, and harvested supernatants were immunoprecipitated with polyclonal anti-HIV-Ig. Bands consistent with gp120 (SU) and p66 (RT) were detected by SDS-PAGE of viral pellets from the fluorescent vectors as well as control non-fluorescent wild-type NL4-3 virus preparations (**Fig. 1C**).

The viral constructs make all viral proteins excepting Nef. Contrary to a previous report [50], we found that while Nef was detectable from a plasmid expression vector and two different wild-type NL4-3 transfections, no expression of Nef was evident from our fluorescent constructs (**Fig. S1**) or from the source constructs for the fluorescent reporter genes, kindly provided Miguel Quinones-Mateu. The likely explanation for this deficit is that splicing is functioning efficiently to the reporter gene, but is not supported to the *nef* exon. It is important to note that since the constructs are isogenic other than the reporter genes, Nef is absent from both constructs. Therefore, any potential effect lack of Nef might exert on replicative capacity is controlled for in dual infection competitions.

To verify that the viruses produced by transfection of pNL4-3-MSS-eGFP and pNL4-3-MSS-DsRed2 are capable of mediating multiple round infections, we harvested virus-containing supernatants from transfected HEK293T cells and used RT activity to normalize the viral content. Constructs containing the wt NL4-3 Env sequence are exclusively CXCR4 tropic. We infected U87.CD4.CXCR4 with equivalent units of RT activity and monitored virus replication kinetics over the ensuing 12 days (**Fig. 1D**). RT activity increased in supernatants from infected cultures of both fluorescent viruses, demonstrating replication competence. The maximum steepness of the sigmoidal curve for eGFP virus was 6.28 CPM/day (90% CI 5.57–6.98), and for DsRed2 virus - 6.93 CPM/day (90% CI 6.16–7.70). Fifty-percent maximal accumulation of RT activity (RT_50_) occurred at approximately Day 6 of culture for both constructs (eGFP virus: 6.20 days, DsRed2: 5.90 days). These values suggest the two fluorescent viruses replicate with near identical replication kinetics despite containing fluorescent reporter genes from two bioluminescent species.

**Figure 2:**
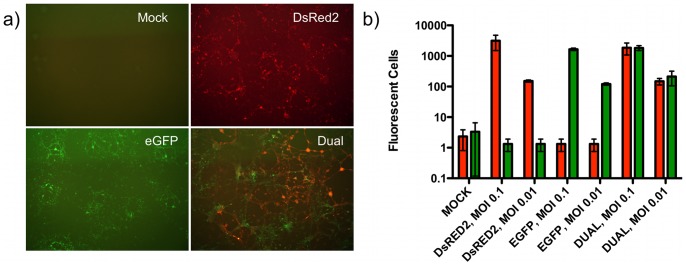
Infectious molecular clones express fluorescent protein reporter upon infection of target cells. **a)** U87.CD4.CXCR4 cells were infected at a MOI of 0.1 with NL4-3-MSS-eGFP, NL4-3-MSS-DsRED2, or both viral variants. Five days post-infection, cells were imaged by fluorescent microscopy (40×). **b)** Fluorescent events from mono- and dual-infection of U87.CD4.CXCR4 cells at MOI of 0.1 and 0.01 were enumerated by flow cytometry. Fluorescent cells were quantified on an Influx cell sorter, excitation 488-nm (eGFP) and 561-nm (DsRed2). MOI of 1.0 was not quantified due cell death in mono- and dual-infections, as well as the presence of dually infected cells (double positive ‘yellow’ cells).

However, evaluating replication kinetics in parallel often fails to detect differences in replication capacity when such differences actually exist. Therefore, to directly and quantitatively test the impact of the distinct reporter genes in the context of the wild-type NL4-3 Env allele, dual-infection competitions between NL4-3-MSS-eGFP and NL4-3-MSS-DsRed2, along with parallel control mono-infections were set up on U87.CD4.CXCR4 cells at multiplicity of infection (MOI) of 0.01, 0.1, and 1.0. Five days post-infection, eGFP and DsRed2 signals were readily visible via fluorescent microscopy (**Fig. 2A**) and infectious events were quantified via flow cytometry. Comparable numbers of infectious events were detected for each fluorescent virus at a given MOI using the 561-nm laser for DsRed2 and 488-nm for eGFP (**Fig. 2B**). We observed a dose-response relationship between the two fluorescent viruses in mono-infections (**Fig. 2B**). Infections at high MOI (1.0) were not analyzed due to extensive cell death in all cultures, and the presence of dually-fluorescent (yellow), and therefore dually-infected, cells in the competitive infections (data not shown). We reasoned that such cultures could exhibit phenotypes resulting from mixed genetics. No difference in fitness between the two wild-type Env viruses in dual-infection competitions was detected at a MOI of 0.1 or 0.01 (**Fig. 2B**) indicating that neither fluorescent reporter gene imparted a significant gain or loss of viral fitness. We were therefore confident that the distinct fluorescent viral vectors would have the capacity to resolve subtle fitness differences in competitive infections.

### 1157i C2-V4 Chimeras

As with other studies of HIV-1 transmission, our previous findings suggested that restricted, or selective, transmission occurred to 1157i. Previous genetic analyses of the C2-V4 region of Env showed that diversification increased over the course of infection. Divergence essentially paralleled diversification, and was associated with accumulation of non-synonymous changes. Nevertheless, tropism for CCR5 was maintained and neither sequence length, or numbers of potential glycosylation, increased with time [60]. The nucleotide changes/month as well as the amino acid changes/month, for a minimum of 23 haplotypes/time-point, are presented in Fig S2. Using co-cultured virus and patient sera, we previously documented that *de novo*, as opposed to passively acquired maternal, neutralizing responses were evident at 12 months, and from thence onward [60]. These humoral responses appeared to correlate with ongoing Env diversification. Moreover, comparative studies of Env diversification utilizing a pathogenic simian-human immunodeficiency virus containing the 1157i envelope sequence (SHIV-1157ipd3N4) have demonstrated common genetic patterns among two species of non-human primates and the original infected infant [62], [63]. One previous study reported a positive correlation between temporal *env* C2-V3 diversification and viral fitness in Subtype B infected adult men [48]. However 1157i is a drug-naïve, slow-progressing infant infected with subtype C HIV-1 *in utero*. Given that it was clear that Env genotypically evolved in a temporal fashion in the patient and in the two primate models, we asked how sequence evolution was impacting the temporal fitness landscape in 1157i. Was Env-mediated fitness increasing or decreasing with variation and selection of C2-V4, or was the region simply undergoing variation without impact on fitness?

Patient-derived C2-V4 HIV-1 *env* sequences were originally amplified from genomic DNA for genetic characterization based on sequencing and phylogenetics [60]. In the current study, archival clones of these C2-V4 sequences in pGEM-T were subjected to amplification with PCR primers that templated the 5′ *Age*I and 3′ *Sbf*I restriction sites compatible with NL4-3-MSS *env* to generate cloneable *env* subgenic fragments (**Fig. 1A**). Twelve individual *env* C2-V4 alleles were amplified from 0, 12, 18, 24, 36, 48 and 67-month pGEM-T constructs (**Fig. S3**). Alleles were selected on the basis of their genetic distance from one another and proximity to the major phylogenetic branch of the subsequent time-point.

### Fitness Quantification in Primary CD4^+^ T Cells

**Figure 3:**
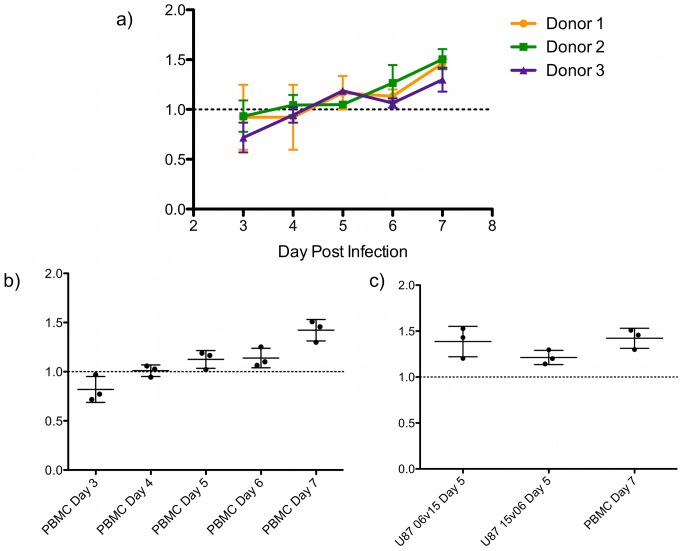
Chimera fitness in PBMC and U87.CD4.CCR5 cells. **a)** Stimulated CD4+ T-cells isolated from three genetically distinct blood donors were infected with chimeric viruses generated from patient viral sequences isolated at birth, NL4-3-00m06-eGFP and NL4-3-00m15-DsRED2. Infected cells were quantified by flow cytometry to calculate relative fitness (W) on days 3–7 post-infection. W of NL4-3-00m15-DsRED2 shown. **b)** Mean NL4-3-00m15-DsRED2 W from each donor (dots) with standard deviation (bars) over time. **c)** Competitions between NL4-3-00m06-eGFP and NL4-3-00m15-DsRED2 and the reciprocal NL4-3-00m06-DsRed2 and NL4-3-00m15-eGFP were repeated in U87.CD4.CCR5 cells. In both competitions, the W of NL4-3-00m15 in U87.CD4.CCR5 at Day 5 post-infection was not statistically different from the W calculated in PBMC at Day 7.

Our data utilizing DsRed2 and eGFP infectious molecular clones containing wild-type NL4-3 envelopes demonstrated equivalent replication kinetics and fitness (**Fig. 1d** **&** **2b**). Sequence analysis of 1157i C2-V4 alleles revealed only a single Ser/Thr polymorphism in a sequon at position 227, (**Fig. S3**) between date-of-birth samples 00m06 and 00m15. Viral stocks of chimeras 00m06 (eGFP) and 00m15 (DsRed2) were generated by transfection of HEK293T cells. We anticipated that competitions between these nearly identical chimeras would produce neutral fitness outcomes. To quantify fitness of these initial chimeras, stimulated CD4+ T-cells were isolated by negative immunomagnetic sorting from three genetically distinct blood donors. The enriched CD4^+^ populations were infected at an MOI of 10 in mono- and dual-infections, a multiplicity sufficient to ensure production of fluorescent cells for fitness calculations without generation of co-infected cells (dually fluorescent). Independent infections were assayed by flow cytometry in triplicate on days 3–7 to quantify eGFP and DsRed2 positive cells for calculation of relative fitness (W) values for the 00m06 and 00m15 C2-V4 alleles (**Fig. 3a**). Fitness is calculated by quantifying the change in the proportion of each competitor (infectious events) in the dual-infection to the quantity of infectious events in matched mono-infections, which act as internal controls. On this fitness coordinate system, maximum fitness or competitiveness is 2.0, whereas total lack of fitness is 0.0, and neutrality is at 1.0. We were surprised to find that competitions between 00m06 and 00m15 alleles resulted in significant fitness differences at the endpoint (7 days post-infection). Initial proportions of infected cells from the dual-infection suggested that 00m15 was less fit than 00m06 (Day 3, W 00m15_DsRed2_ = 0.8197±0.1321) (**Fig. 3a, b**). However, 00m15 fitness value steadily increased over the course of 7 days indicating that the initial findings resulted from titer underestimations for the innocula used to set up the dual- infection that required 00m15 to undergo multiple rounds of replication in both the mono- and dual-infections before demonstrating competitive exclusion. The increase in 00m15 fitness occurred in all blood donors, and there was no difference in the slope of the increase in fitness in the three donors. When the mean fitness value from all three donors was plotted over time, at Day 7, 00m15 was more fit than 00m06 (W 00m15_DsRed2_ = 1.422 vs. W 00m06_eGFP_ = 0.578±0.1094) (**Fig. 3b**). These data, from viruses differing by a single, seemingly conservative, point mutation in a maintained N-linked glycosylation motif, demonstrate that this fitness quantification platform is potentially more sensitive than those previously reported.

### Fitness Quantification in U87.CD4.CCR5 Cells

The number of dual infections and controls required to analyze fitness for all chimeras precluded the use of primary cells as targets for infection. Moreover, though we saw no differences between the relative fitness values calculated from three different blood donors, the use of a defined cell line, such as U87.CD4.CCR5, previously utilized for competition assays, would simplify the assay in terms of time and cost as well as reducing the potential introduction of donor-specific effects, such as variation in receptor/coreceptor expression levels [46]. U87.CD4.CCR5 cells were infected at an MOI of 0.1 to determine whether they recapitulated fitness outcomes in PBMC. By Day 5, both the 00m06 (eGFP) *versus* 00m15 (DsRed2) and the reciprocal infections, 00m06 (DsRed2) *versus* 00m15 (eGFP) reproduced the same fitness relationships determined in PBMC (W 00m15_DsRed2_ = 1.213±0.07732, 00m15_eGFP_ 1.386±0.1659) (**Fig. 3c**). Competitions in U87.CD4.CCR5 using independently generated and titered viral stocks not only recapitulated outcomes from PBMC dual-infections, but did so in a shorter amount of time and at a lower MOI. Therefore, subsequent analyses were carried out in U87.CD4.CCR5 at Day 5 to ensure that infectious events were readily detectable, and that the more competitive variant had not competitively excluded the less fit variant to the point of extinction.

**Figure 4:**
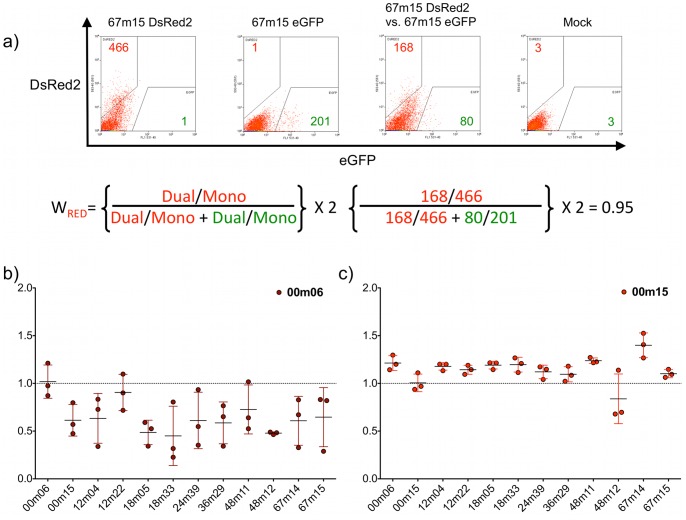
Results from individual patient chimera competitions. **a)** U87.CD4.CCR5 cells were infected with NL4-3-67m15-eGFP, NL4-3-67m15-DsRED2 at a MOI of 0.1 in mono- and dual-infections. The numbers of fluorescent events were enumerated by flow cytometry. The numbers of events were then used to calculate relative fitness (W) values. This process was carried out for all competitions, in triplicate. **b)** The resulting W values from all NL4-3-00m06-DsRED2 vs. all other viruses in eGFP, and **c)** NL4-3-00m15-DsRED2 vs. all other viruses in eGFP. Circles represent individual W values, black bars represent means, and error bars represent standard deviation. Results from all chimeras in **Fig. S4**.

Virus stocks for all chimeras were produced by transfection of HEK293T cells, titered and subjected to triplicate, dual-infection competitions versus all other chimeras in the opposite color vector, including itself. Each chimera was also introduced into the alternative fluorescent vector and subjected to reciprocal competitions. Relative fitness (W) values for each competition were determined in comparison to parallel mono-infections with each chimera at the same MOI. The self-self competitions, which are theoretically anticipated to be 1.0, were not experimentally exactly 1.0. An example competition, where the DsRed2 and eGFP fluorescent reporter viruses both contain the C2-V4 sequence haplotype 15 from 67 months post-infection (67m15), is shown in **Figure 4A**. This example demonstrates the anticipated outcome that is competitions of the same Env sequence against itself results in a neutral outcome W ∼1.0 (0.99±0.31). While all competitions performed at the same time with the same stocks of virus were comparable to one another, to support comparison between all chimeric competition sets, all other competitions in each data set were adjusted to reflect the deviation between theoretical (1.0) and actual values for the self-self competitions. For example, 67m14 self-competitions resulted in a W of 1.13±0.29 (n = 3, ± SD), slightly higher than the expected 1.0. To adjust the values to 1.0, the self-values were multiplied by a factor of 0.927, thus the adjusted W was 1.05±0.27. Similar adjustments for self-self variation were carried out for all chimera competition sets. All self-self competitions had a mean of 1.03 (95% CI 0.98–1.1), which was not statistically different from 1.0 (One sample *t* test).

When chimeras were individually analyzed, we identified *env-*chimeras that were consistently less competitive, W<1.0, (Losers); whereas other *env* sequences imparted a more competitive phenotype W >1.0 (Winners). Disparate fitness phenotypes were quantified among *env* sequences isolated at the same time-point (**Fig. 3** **&** **4B, C**). Thus the fitness assay readily discriminates *env* C2-V4 sequences that impart greater or lesser fitness on a common viral genetic background, and can do so within the spectrum of polymorphisms contained within a single individual or within a single time-point of collection.

**Figure 5:**
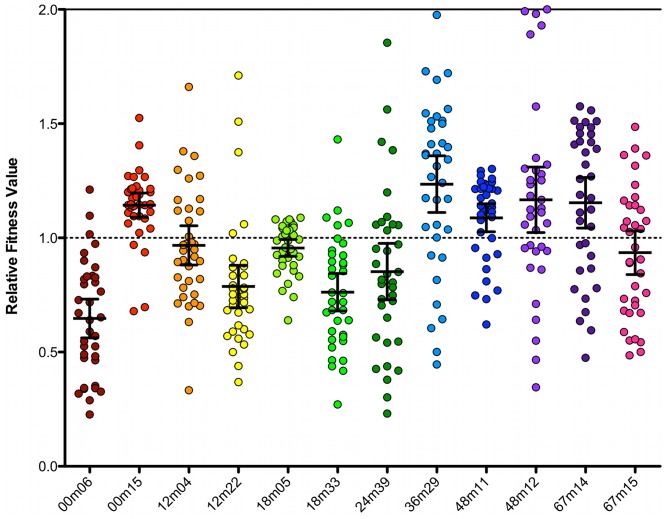
Aggregate data from all patient chimera competitions. Each C2-V4 patient chimera (DsRed2) was competed against all others (eGFP) in triplicate. W_RED_ was calculated for each competition, and plotted in aggregate for each chimera. Black bars represent mean, and 95% CI.

Aggregate analyses of relative fitness values from all competitions (n = 396 dual infections, associated eGFP and DsRed2 mono-infections, and mock infections) are presented in **Fig. 5**, with circles representing individual data points, black bars representing means, and error bars representing 95% CI (numeric data **Table S1**, individual competitions **Fig. S4**). In individual competitions between chimeras (**Fig. S4**), we observed a wide range of W values demonstrating that the system has discriminatory capacity to segregate winners and losers in individual competitions (range = 0.23–2.0). With the exception of 12m04 and 67m15, all average fitness values were significantly different from neutrality (95% CI), suggestive of a rugged fitness landscape (**Table S1**). From **Figure 5** **& Table S1**, it is also clear that there were chimeras that were significantly more fit than others (e.g. 36m29). Equally, there were those that were significantly and consistently, less fit (e.g. 00m06). In many cases, however, relative fitness values were statistically indifferentiable from one another (**Table S3**). Taken together, the samples of C2-V4 sequences derived from this infected individual imparted average viral fitness values that clustered about neutrality (Range: 0.65–1.24, Mean: 0.97, Median: 0.96) (**Fig. 5** **–** black bars). While diversification and divergence were increasing with time in this region of Env, no statistically significant temporal trend in fitness outcomes could be detected.

In an effort to detect temporal fitness trends in C2-V4 we combined the outcomes of all fitness competitions into categories based on allele isolation time-point (e.g. data from 00m06 and 00m15 were combined into a 00m data set). Analysis of the temporally grouped competition data sets resulted in a linear regression slope that was not statistically differentiable from zero, supporting the concept of a flat fitness trajectory over time (data not shown). These data suggest the fitness distribution between time-points needs to be sampled at a greater depth, and that more *env* sequences from more patients with different courses of disease need to be longitudinally analyzed using this method to determine whether evolutionary trajectories are evident.

### Biochemical Characterization of 1157 V3-loop Chimeras

The HIV-1 envelope glycoprotein carries out several essential processes in HIV-1 replication that can be individually quantified. Given the lack of a clear temporal relationship with fitness we sought to investigate how viral fitness associated with biological functions of Env. We tested for relationships between fitness and parameters of Env function, including synthesis and processing of gp160, incorporation of Env trimers into virions, Env affinity for CD4 and CCR5, and single-round virus entry.

### Env Synthesis, Processing, and Virion Incorporation

**Figure 6:**
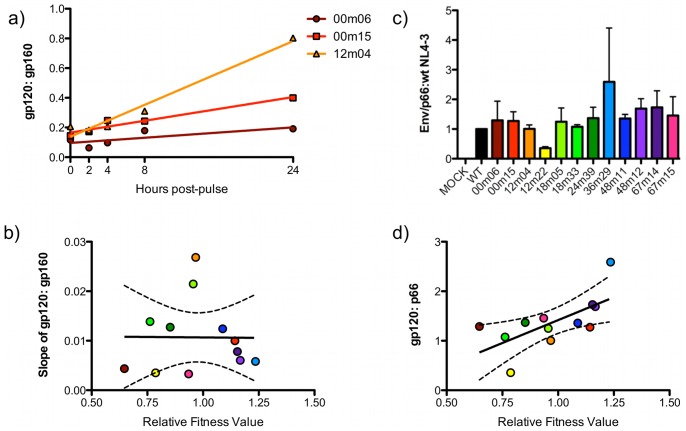
Patient chimeras differ in Env processing and incorporation. **a)** 293T cells were transfected with patient eGFP chimeras. 24-hours post-transfection, cells were radiolabeled with ^35^S. Cells were lysed at the indicated time-points, immunoprecipitated with HIV-Ig, and resolved by SDS-PAGE in order to quantify the processing of gp160 and accumulation of gp120. To quantify the processing of Env, the slope of the ratio of gp120 to gp160 verses time was calculated for each chimera (00m06, 00m15, 12m04 shown). **b)** Slope values were plotted against average relative fitness values to determine correlation (dashed lines 95% CI). **c)** Supernatants from radiolabeled 293T patient eGFP chimeras’ transfections were pelleted, lysed, and immunoprecipitated with HIV-Ig. SDS-PAGE was used to resolve gp120 and p66 protein bands for calculating incorporation of Env relative to wild-type virus, in triplicate. **d)** This ratio was plotted against relative fitness values, and revealed a modest positive correlation (R^2^ = 0.4349) (dashed lines 95% CI).

Using metabolic labeling and immunoprecipitation, followed by quantification of signal using a phosphorimager, we observed differences between chimeras in the rate of change of the gp120:gp160 ratio (i.e. the processing of gp160 into gp120) over the course of a 24-hour chase (range of slopes = 0.0035–0.027 DLU/hour) (**Fig. 6A**). Despite these differences, the efficiency of processing did not correlate with average fitness values (**Fig. 6B**). However, when the level of gp120 incorporated into pelleted virions, relative to p66 RT, was investigated (**Fig. 6C**), there was a modest correlation with average fitness values (R^2^ = 0.43) (**Fig. 6D**). This suggested that perhaps quantity, rather than quality of Env was playing a role in determining fitness. However, despite obvious differences in levels of Env synthesis and processing as well as the efficiency with which Env was incorporated, neither biosynthetic parameter appeared solely predictive of Env-mediated fitness.

### Viral Entry

**Figure 7:**
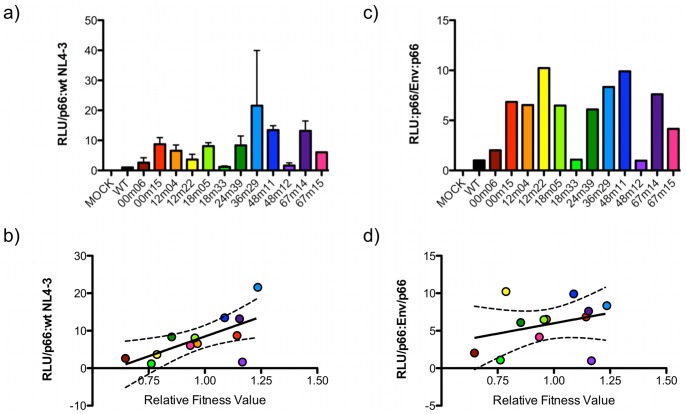
Chimera infectivity has a modest correlation with fitness, while infectivity per incorporated Env does not. **a)** Infectivity of each chimera was assayed via luciferase activity of TZM-bl cells (Relative Light Units, RLU), relative to p66, as determined by immunoprecipitation of radiolabeled virions (see **Fig. 5c**), relative to wild-type NL4-3, in triplicate. **b)** The average infectivity value was plotted against average relative fitness values, and revealed a modest correlation (R^2^ = 0.4448) (dashed lines 95% CI). **c)** The ratio of average infectivity to average incorporation of Env was calculated to determine average infectivity per incorporated Env. **d)** When this ratio was plotted verses average relative fitness values, there was no correlation (R^2^ = 0.1058) (dashed lines 95% CI).

As anticipated from previous publications [45], [46], there was a positive trend between Env-mediated infectivity (**Fig. 7A**) and average relative fitness values (**Fig. 7B**). However, this trend exhibited only a moderate correlation (R^2^ = 0.44), with two data points falling outside of the 95% confidence range. This indicates that while there may be a relationship between Env, infectivity, and viral fitness, one cannot confidently predict the relative fitness of a virus from normalized single-round infectivity results. This is especially true with small sample sizes typical of experiments with human samples. The correlation was lost when co-adjusted for the level of Env incorporation into particles [infectivity (DLU)/p66)/incorporation (Env/p66)] (R^2^ = 0.11), again suggesting that the fitness trend associated with Env was influenced by the quantity of Env on the surface of virions, rather that the functional quality of individual Env chimeras (**Fig. 7C–D**). Together, these results suggest that fitness imparted by Env might be more a function of avidity, resulting from multivalent low affinity interaction between Env and the target, than from high affinity, but low abundance, interactions.

### Receptor and Coreceptor Affinities

**Figure 8:**
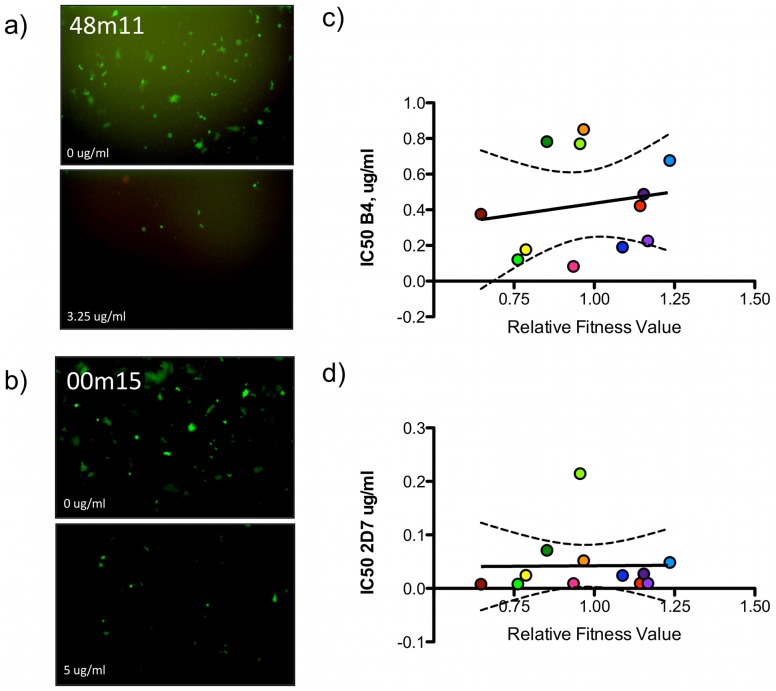
Chimera affinity for CD4 or CCR5 is not correlated with fitness. Viral stocks of eGFP chimeras were incubated with serial dilutions of a CD4 competitor (B4 Ab) or CCR5 competitor (2D7 Ab), and added to TZB-bl indicator cells. Though reduction in eGFP expression with increased competitor antibody could be visualized via microscopy **(a, b)** (40×), IC_50_s were calculated from luciferase activity assays, in triplicate. When IC_50_ values were plotted against average relative fitness values, there was no correlation for CD4 affinity (**c**) (R^2^ = 0.02970) or (**d**) CCR5 affinity (R^2^ = 0.0001508) (dashed lines 95% CI).

To begin to test relationships between avidity or affinity and fitness, we next evaluated parameters associated with viral entry into a new host cell for their correlation with fitness. Affinity of each chimeric Env for the primary receptor, CD4, was assayed via competition with the anti-CD4 antibody B4 for available receptors on TZM-bl cells (**Fig. 8A**). The introduction of the subtype C C2-V4 sequences converted NL4-3 from exclusive X4 tropism to R5 tropism. Therefore, CCR5 coreceptor affinity was assayed by competition with antibody 2D7 (**Fig. 8B**). Despite a wide range of phenotypes (IC_50_ = 0.083–0.85 µg/ml B4, IC_50_ = 0.0079–0.21 µg/ml 2D7), there was no correlation between affinity for CD4 or CCR5 and fitness (**Fig. 8C-D**). In sum, the biochemical quantifications of Env function suggest that relative fitness values are determined by quantity of Env in particles, rather than the affinity of Env for relevant cellular receptors.

## Discussion

### Dual-infection Competitions and Limitations of Current Methods

Links between HIV-1 fitness and disease progression, virus transmission, drug resistance, immune escape, and global epidemiology have been experimentally established. Because of these associations, numerous assays have previously been developed for measuring HIV-1 fitness *in vitro* [24], [26], [47], [64]–[69]. Large differences in fitness can be identified via differential viral growth kinetics [15]. However, these approaches are often incapable of elucidating subtle fitness differences between HIV-1 variants as might be anticipated to exist in genetically related viruses derived from a patient over the course of infection, or in samples from a quasispecies at a single time-point [15], [45], [46], [48].

When two or more variants are in competition with one another, the ‘more fit’ variant will eventually outgrow ‘less fit’ variants by competitive exclusion i.e. ‘survival of the fittest’ as resources become limiting [3], [70]–[72]. In the absence of gross replicative defects, two HIV-1 variants can have virtually identical *in vitro* growth kinetics, yet disparate fitness values in competition with one another [46]. Viral fitness is therefore optimally defined by assaying replication where viruses are competing in an identical environment (dual-infection), and where the performance in the dual-infection can be directly normalized to the replication of each competitor in monoinfections innoculated with the same quantity of virus [49]. In addition to providing a standard for determining proportional changes in viral species in competition, mono-infection controls eliminate concern about the absolute equality of the titers of the two species at the beginning of the competitions.

Heteroduplex tracking assays (HTA) or qRT-PCR have previously been used to quantify HIV-1 fitness in dual-infection competitions among viruses in HIV-1 panels [45], [48], [73], [74]. One of the striking findings was that HIV-1 fitness, in the absence of treatment, is a function of the envelope glycoprotein [46]. While previous methods to quantify fitness have proven unquestionably useful, they are limited by inability to discriminate variants with either too much or too little genetic diversity within the detection target sequence. Likewise, recent methods employing precise parallel allele-specific sequencing have the advantage of precise quantification of fitness differences but require prior knowledge of the sequences under selection as well as specialized instrumentation and expertise [75].

### Advantages of the New Vectors for Fitness Analyses and Other Applications

The infectious molecular clones we describe here contain either the eGFP or DsRed2 fluorescent reporter gene, and, being derived from a previously validated fitness analysis system, provided a simple and direct platform for quantifying Env effects on HIV-1 fitness. A primary advantage of our system over other HIV-1 fluorescent reporter vectors [50] is the inclusion of sites to facilitate precise molecular cloning of *env* genes or subdomains (**Fig. 1A**). The fluorescent signal produced upon viral infection supports quantification of infectious events in dual-infection competitions by universally accessible methods (flow cytometry or qRT-PCR), which in turn, allows for the calculation of relative fitness values. Because the fluorescent reporter is contained within the viral genome, the resulting viruses can be used to quantify infectivity of patient Envs on any target cell type, including primary cells (**Fig. 3**).

These constructs have additional applications. First, co-infected cells skew interpretation of fitness assays due to the capacity for recombination between the competing strains [76], [77]. A substantial benefit of using eGFP and DsRed2 reporters is that they provide the ability to detect co-infected cells (‘yellow’ fluorescence), resulting from high MOI infections. Moreover, since the eGFP and DsRed2 genes are derived from different species (the jellyfish *Aequorea victoria* versus the sea anemone *Discosoma striata*) [78], [79], they are less likely to undergo recombination. Given the MOI we employed, we rarely detected yellow, co-infected cells at the endpoint analysis, and when they were detected, the dual-infection competitions were repeated at reduced MOI. On the other hand, our system readily supports the identification, and cell sorting, of co-infected cells that could be used to investigate the biology associated with HIV-1 recombination.

In addition, minimal spectral overlap in the excitation and emission of eGFP and DsRed2 (excitation 488-nm and 531-nm, emission 509 nm and 582 nm, respectively), limits the need for compensation adjustments and simplifies data acquisition and analysis by flow cytometry. Dual-infection competitions can also be readily quantified via qRT-PCR using amplicons directed against the distinct fluorescent reporters due to their lack of sequence homology [78], [79]. Using this approach, entire collections of *env* chimeras could be detected with the same optimized primer-probes, rather than requiring discrete reagent sets for each individual allele. We have shown that qRT-PCR amplicons directed towards the fluorescent genes have virtually no cross-reactivity towards the other template (**Fig. S6**), making them ideal multiplex RT-PCR targets. Furthermore, as measuring fluorescence *in situ* does not require cell lysis, given cell-sorting capacity in biocontainment, these fluorescent markers support live sorting of infected cells for use in any number of downstream applications. For example, the constructs may prove useful for screening neutralizing antibodies where the restriction sites allow for the introduction of a complete Env ectodomain that could be followed by reductive introductions of smaller sequences to localize Ab binding.

### Longitudinal Ab-driven Divergence of C2-V4 in 1157i

We chose to demonstrate the utility of our system using genetically characterized Env C2-V4 sequences derived longitudinally from a slow-progressing, subtype C HIV-1 -infected, Zambian patient, 1157i. We selected this panel of Env variants for four reasons: 1) because longitudinal variants from a single infant had not been previously evaluated for differences in Env-mediated fitness by dual-infection competition, 2) because we reasoned that these isolates would test the capacity of the assay system to quantify fitness values among closely related sequences, 3) phylogenetic analyses of Env C2-V4 suggested that *env* diversification was taking place at both the nucleotide and amino acid level (slope = 0.25 nucleotide differences/month, 0.18 amino acid differences/month **Fig. S2**) moreover, positive selection was taking place since increases in non-synonymous changes (*d*N/*d*S) over time were detected [60], and 4) immunological assessment of 1157i plasma samples suggested that NAb were exerting selective pressure on Env, since sera collected at later time-points were capable of neutralizing viruses isolated at earlier time-points, but minimally neutralized contemporary viruses. [80]. Collectively these previous analyses suggested that diversification within 1157i *env* had taken place in response to humoral immune pressure.

### Subtle 1157i C2-V4 Variants from the Date of Birth Exhibit Fitness Differences

The C2-V4 region of Env plays a critical role in viral tropism and transmission, as well containing a principal neutralization determinant, the V3-loop [55], [81]. To investigate relationships between viral fitness and sequence diversification in C2-V4, viral chimeras were created by introducing 1157i C2-V4 sequences, isolated from birth to 67 months, into the NL4-3-MSS-eGFP and DsRed2 vectors. Though this region underwent genetic diversification over time in 1157i (without alteration of coreceptor usage), C2-V4 sequences obtained at birth (00m06, 00m15) were virtually identical (**Fig. S3**). This limited DNA diversity only manifested in a single amino acid difference, a Ser or Thr at amino acid 227 in the third position of a potential N-glycosylation site. With such limited amino acid differences between the two variants, one would anticipate that both variants would have similar relative fitness values. However, 00m15 (Thr_227_) proved to be more fit than 00m06 (Ser_227_) in CD4^+^ T-cells isolated from three different blood donors and in U87.CD4.CCR5 cells (**Fig. 3**). We hypothesize that threonine supports preferential glycosylation [82], or perhaps the differential glycan addition to the associated Asn. The resulting altered Env structure and function, thereby contributes to the fitness differential [82]. It is also conceivable that one or the other sequon variants is simply not recognized as a site for glycan addition in the Env folding pathway within the ER. This *in vitro* fitness advantage of 00m15 appears to correlate with an *in vivo* fitness advantage, since the ‘more-fit’ Thr polymorphism was maintained at subsequent time-points. Maintenance of Thr is also consistent with its approximately 2-fold preferential utilization in N-linked glycosylation motifs [82]. The outcomes of competitions between 00m06 and 00m15 chimeras highlight several important points: 1) though *in vitro* fitness assays cannot perfectly mirror the selective environment occurring in a patient, our observations demonstrate that *in vitro* fitness assays yield biologically relevant information, 2) the competition system has the ability to reproducibly differentiate between fitness ‘winners’ and ‘losers’ in primary CD4+ T-cells as well as U87 cell lines, 3) fitness differentials can be identified even when the sequences in question differ by extremely subtle polymorphisms, 4) such information could inform further study of envelope structure and function in relation to viral fitness or other parameters.

### C2-V4 Variants from Later Time-points Suggest Increasingly Complex Fitness Relationships

U87.CD4.CCR5 cells were used for dual-infection competitions pitting each chimeric Env virus against all others, in what was essentially a viral ‘round-robin’ tournament. This cell line was utilized in lieu of PBMC because of the substantial number of competitions and controls for fitness analysis in this study, and because the U87.CD4.CCR5 cell line has been used in previous fitness analyses [46]. We showed that it not only reproduced PBMC outcomes, but did so with less virus (lower MOI) in a shorter amount of time (**Fig. 3**). Relative fitness values from each individual competition were calculated and ranged between 0.23-2.00 (**Table S1**).

When analyzed individually and in aggregate, head-to-head competitions yielded complex findings. Though we compared sequences from a relatively small region of Env, derived from the same patient and from within time-points, identifying molecular determinants of fitness was not as simple as identifying ‘more-fit’ and ‘less-fit’ amino acids at set locations. Shared polymorphisms could be associated with a ‘more-fit’, ‘less-fit’, or ‘neutral’ outcome, depending on their context with other polymorphisms within a given allele. For example, 12-month time-point sequences, 12m04 and 12m22, like those from 00m, differed from one another by a single amino acid that resulted in loss or retention of a putative glycosylation site (N or S at amino acid 329 **Fig. S3**). This polymorphism did not have an effect on viral fitness in head-to-head competitions, nor was there a statistical difference between 12m04 and 12m22 average relative fitness values (**Fig. 5** **, Fig. S4, Table S2**). Based on our finding that 12m22 had a neutral or less fit outcome in average relative fitness values versus eleven other alleles, and the fact that, S_329_ was not maintained in subsequent time-points makes it attractive to conclude that S_329_ was not a fitness-favorable polymorphism. However, the exception appeared at the 48-month time-point, with 48m11 containing N_329_ and 48m12 the S_329_. Despite an additional 26 polymorphisms between 48m11 and 48m12, these two viruses were neutral in head-to-head fitness, and did not have significantly different average fitness values (**Fig. 5** **, Fig. S4, Table S2**). However, the additional 17 polymorphisms between 12m22 and 48m12, both of which contain the Ser_329_, lead to 48m12 being more fit in both head-to-head and total average fitness. In fact, the S_329_ containing 48m12 had significantly higher average fitness values than 5 of the other 11 variants, but was not detected at 67m. The genetics of the subsequent time-point along with our fitness results, lead us to hypothesize that the S_329_ is either a minor variant, a neutral polymorphism in this context, or it is actively negatively selected against by a factor not directly measured (i.e. antibody selection). In the future, resolving the complexity in the relationships between genetic polymorphisms and fitness outcomes would require more thorough sampling at a given time-point as well as comparison to both prior and subsequent time-point allele frequencies and associated fitness values.

Our results also highlight a benefit to competing all viruses against one another, as opposed to competing the chimeras against reference strains. A viral ‘round robin’ does not assume that 1) all fitness results are transitive (a>b, b>c, therefore a>c), or 2) all fitness results are cumulative (a>b by 0.1, b>c by 0.1, therefore a>c by 0.2). While these assumptions might be valid in some circumstances, they would have been incorrect assumptions for this study. For example, chimeras 12m04 and 18m33 were approximately neutral in head-to-head competition with one another (**Fig. S4**) (12m04_DsRed2_ vs. 18m33_eGFP_ = 0.95±0.13, 12m04_eGFP_ vs. 18m33_DsRed2_ = 1.0±0.06), and their average relative fitness values were not statistically different (**Table S2**). Competition against reference strains would assume that these chimeras would fare similarly against others, winning, losing, and tying against the same viruses by the same magnitude. That was not the case. Chimera 12m04 had a statistically higher average relative fitness than 00m06, but lower than 36m29. Average relative fitness of 18m33 was lower than 00m15, 36m29, 48m11, 48m12, and 67m14 (**Table S2**). So while the amino acid variation between 12m04 and 18m33 appeared to have no impact on viral fitness in head-to-head competition, the true impact of that variation became apparent when the viruses were competed against other alleles. The ‘round robin’ strategy made possible by our competition system allowed us to elucidate an enriched fitness landscape that would have remained hidden had reference strains been used.

### Extinction was not Observed

Fitness competition data was further analyzed by grouping all competition outcomes for each chimera. Previous fitness analyses, particularly those between subtype B and C, revealed substantial disparities in replicative fitness between subtypes. In those instances, it was necessary to dilute ‘more-fit’ subtype B variants by orders of magnitude to prevent complete exclusion of the ‘less-fit’ subtype C variant [45]. Dilutions were not necessary for the competitions in this study, as no variant was grossly more or less fit than the others over the time course of the analysis. This is likely because all variants were derived from a single individual. An alternative reason could be that no critical Env fitness determinants lie inside the C2-V4 region of Env. The latter explanation is not supported by data from previous studies that have demonstrated the essential nature of the V3-loop [28], [51]–[59]. Our data shows that competitive exclusion is taking place and that given sufficient time, extinction would occur (**Fig. 3**). However, allowing such complete exclusion reduces complex fitness assays from quantitative measures to less informational binary results.

### Potential Relationships between Humoral Responses and Fitness Outcomes

While there were statistically significant differences between average relative fitness values, none of the chimeras were a universal ‘winner’ or ‘loser’. The values clustered between 0.65 and 1.23, suggesting that in spite of sequence diversity in C2-V4, the chimeras have similar average fitness (**Table S1–S2**). Our previous neutralization analyses with 1157i revealed an evolving humoral neutralizing response that acted against previous viral isolates but not against contemporaneous isolates. That is, 48m sera neutralized virus co-cultured from all previous time-points but had little impact on the 48m or subsequent 67m isolate. Those results along with the increases in nonsynonymous changes in C2-V4 implied the host antibody response was acting to select the viral population in this region of Env. Plasma samples from 1157i were exhausted in previous analyses [60]. Thus, we were unable to directly assess relationships between replication competitiveness and neutralization sensitivity of chimeric Env alleles. As such, our results represent quantification of differential replication capacity in the absence of demonstrable selection.

However, in the Amsterdam cohort of HIV-1 subtype B infected adults, the impact of neutralizing antibody, and even broadly neutralizing Ab on autologous virus growth kinetics, as a surrogate for fitness, was tested. No inverse relationship between fitness and neutralization Ab titer or breadth was reported [83], [84]. Similarly, Troyer *et al*. demonstrated that Env CTL escape variants suffered a fitness deficit less frequently than escape variants in other structural genes [85]. And, mutations in Env that block recognition by NAb have also been shown to have little impact on viral fitness [83], [84]. Recent findings have suggested that broadly neutralizing antibodies may select Env quasipecies resulting in reduced viral fitness [75], [86], [87]. These effects appear to be temporary since viral load in many cases appears to rebound [86].

We have no evidence that 1157i possessed bNAb and given the time post-infection required to develop bNAb responses, it seems unlikely [88]. But it is reasonable to speculate that neutralizing antibodies with more restricted breadth have less impact on fitness than do bNAb, resulting in a pattern similar to that observed in 1157i. This concept requires further experimental validation but can be inferred from the finding that only some of the escape variants generated in early infection undergo a fitness deficit as a result of humoral selection [87]. Collectively, published data, and that from the current study, suggests that HIV-1 Env, tolerates mutation leading to immunological escape while maintaining protein functionality and viral fitness or replication capacity at adequate levels.

### Env Quantity/avidity Measures Trend Most Closely with HIV-1 Fitness

In an effort to understand how fitness is maintained in the face of genetic diversification, we biochemically characterized the functions of Env in the HIV-1 replication cycle, including synthesis and processing, incorporation into virions, affinity for CD4 and CCR5, and infectivity. As expected from previous reports, infectivity did positively trend with average fitness values [73]. However, infectivity was not statistically useful for predicting relative fitness (R^2^ = 0.44) (**Fig. 7B**), similar to what others have recently reported [75]. Similarly, incorporation of Env into virions contributed to viral fitness since a positive trend between incorporation and relative fitness (R^2^ = 0.43) was detected (**Fig. 6D**), a weak correlation previously described by others [43]. Again, however, incorporation levels are not a useful proxy assay for estimating fitness, as other studies have not found statistically significant differences in Env incorporation levels in pseudoviruses generated from elite suppressors and chronic progressors [89]. The ratio of infectivity per Env ((Infectivity/p66)/(Env/p66)) could be considered the efficiency of a given quanta of Env to support entry. This ratio, however, was not correlated with average fitness (R^2^ = 0.10) (**Fig. 7D**).

Interestingly, several chimeras contained polymorphisms in putative CD4 contact residues in C2 and C3, which have previously been associated with higher or lower CD4 affinity [90]. Yet despite the finding that chimeras exhibited a wide range of affinities for CD4 (B4 IC_50_ 0.083–0.85 µg/ml), and CCR5 (2D7 IC_50_ 0.0079–0.21 µg/ml), neither receptor binding parameter correlated with average fitness (R^2^ = 0.033, 0.00035 respectively) (**Fig. 8C-D**). This suggests that while there clearly must be some affinity between Env and CD4 as well as with coreceptor, a range of affinities can be tolerated without adversely effecting viral fitness. Collectively, these results support the conclusion that it is Env quantity, and perhaps avidity, rather than affinity that most potently influences virus entry and therefore fitness.

In the correlations between biochemical assays and fitness, no chimera was consistently an outlying data point, above or below the 95% CI. No chimera was shown to consistently over- or under-perform and skew the correlations. One interpretation of these data is that there are multiple pathways to maintaining fitness adequacy in the Env-mediated HIV-1 entry pathway. An examination of functional assay outliers highlights this basis of this concept. For example, 36m29 and 48m12 both had similar fitness values statistically greater than 1.0. In functional assays, however, these chimeras demonstrated distinct biochemical properties, including viral entry (**Fig. 5** **&** **7**). Previous studies investigating the biochemical properties of transmitted Env variants determined a wide range of phenotypes [91]. Likewise, our results suggest that fitness, while determined by the HIV-1 entry process, is defined by the sum of the efficiencies with which Env is able to accomplish each step, suggesting that no single readout is likely to satisfactorily correlate with fitness. It also implies that each parameter assayed makes an important, but not necessarily equal, contribution to the entry process and therefore to fitness. Thus, no functional assay is able to serve as a proxy measure for fitness.

### Env C2-V4 Fitness Landscapes and Population Biology Concepts

Population biology has supported the concept of “survival of the fittest” suggesting that natural selection favors the propagation of genomes that impart a pinnacle of fitness to the organism (fitness peaks) and deviation from those sequences results in a drop in overall fitness (fitness valleys). In such a model, populations with high mutation rates, such as retroviruses, would lack the capacity to maintain genome integrity should they reach a fitness peak, and therefore would fall into a valley. Alternatively, a ‘flat’ or ‘mutationally robust’ fitness landscape can be modeled in two dimensions as a broad plateau of sequence space populated by a host of nearly equivalent variants [41], [42] that is, a population with adequate replicative capacity despite substantial degrees of sequence polymorphism. Our data, derived from *in vitro* measurements of patient-derived sequences, supports aspects of both of these models. The restricted variation between average relative fitness values among chimeras supports the concept of a flat fitness landscape within *env* in this patient. The resolution of this landscape is limited, and more robust sampling and fitness characterization at each time-point would further elucidate the topography of the fitness landscape within this or other patients. However, samples collected from any individual patient, even at disparate time-points, occupy a very limited portion of the sequence space being accessed by the global HIV-1 population, and in each individual, the sequence space explored in Env is localized around different fitness ‘heights’. It is possible that there are defined peaks and valleys for fitness within *env* as a global population, but again, more robust sampling and fitness characterization would be required to resolve the fitness/replicative capacity topography of *env*. It is also possible that different fitness topographies apply to different HIV-1 gene products due to the distinct and diverse selective pressures acting on those proteins, and it is equally conceivable that different topographies might apply to different domains of Env for the same reason. The current analyses are admittedly limited by the sequence content available for analysis, but nevertheless reveal a remarkable fitness landscape for a functionally critical region of Env.

What fitness topography is being explored by HIV-1 in Env can only be resolved if more sequences, and more complete *env* alleles are subjected to competitive fitness determinations, pitting isolates against closely related sequences and against those inhabiting distant regions of sequence space. Here we have developed a system that makes quantification of fitness by dual infection competition readily implementable, as well as providing a tractable system for quantitatively evaluating various aspects of HIV biology, or HIV-1-immune response interactions.

### Conclusions

To quantitatively evaluate HIV-1 fitness, we generated novel chimeric HIV-1_NL4-3_ infectious molecular clones containing patient 1157i C2-V4 env sequences, which co-expressed either eGFP or DsRed2 fluorescent reporters upon infection. While the system was capable of differentiating ‘winners’ and ‘losers’ in individual competitions with exceptional sensitivity, no chimera was a universal ‘winner’ or ‘loser’ and fitness phenotypes were not correlated to a single Env-mediated step in virus entry. Within the limits of the sample set, the apparent longitudinal pattern of fitness evolution presented as a flat, near neutral, distribution; one consistent with the concept of selection of C2-V4 for mutational robustness.

We conclude that there are multiple pathways to adequate HIV-1 fitness in Env, but there appears to be no surrogate marker for HIV-1 fitness; it is optimally quantified by dual-infection competition.

## Methods

### Patient 1157i Samples

The Env sequences subjected to analysis here were genetically characterized previously [60]. Peripheral blood mononuclear cell (PBMC) DNA samples were collected from a child infected with HIV-1 (subtype C) *in utero* in Zambia, Africa, infant 1157 (1157i). This child underwent limited progression to AIDS without the use of antiretrovirals, providing the opportunity to investigate the longitudinal evolution of Env.

### Cell Culture

Human embryonic kidney (HEK293T) cells, obtained from ATCC, and HeLa indicator cells (TZM-bl), obtained from the NIH AIDS Research and Reference Reagent Program, Division of AIDS, NIAID, NIH: from Dr. John C. Kappes, Dr. Xiaoyun Wu and Tranzyme Inc. [92], were maintained in Dulbecco’s modified Eagle medium (DMEM) supplemented with 10% FBS, L-glutamine (20 mM), penicillin/streptomycin (100 mg/ml) (Gibco). Astroglial cells expressing CD4 and CXCR4 (U87.CD4.CXCR4), and CD4 and CCR5 (U87.CD4.CCR5) were obtained from the AIDS Research and Reference Reagent Program, Division of AIDS, NIAID, NIH from Dr. Hong Kui Deng and Dr. Dan R. Littman [93] and maintained in DMEM supplemented with 15% FBS, L-glutamine (20 mM), penicillin/streptomycin (100 mg/ml), puromycin (1 µg/ml), G418 (300 µg/ml) (Gibco).

Primary blood mononuclear cells (PBMC) were isolated from HIV-seronegative blood donors via Ficoll-Hypaque density gradient centrifugation (Oklahoma Blood Institute). PBMC were activated with PHA (5 µg/ml, Sigma) for 48 hours, and then washed thoroughly in PBS. Untouched CD4+ T-cells were isolated utilizing Miltenyi CD4+ T-Cell Isolation Kit II (#130-091-155) and AutoMACS according to manufacturer instructions. Purified CD4+ T-cells were resuspended in PBMC growth media: RPMI 1640 supplemented with 10% FBS, L-glutamine (20 mM), penicillin/streptomycin (100 mg/ml), and IL-2 (10 U/ml, Roche).

### Plasmid Construction

Fluorescent protein genes from pNL4-3-Δenv-eGFP and pNL4-3-Δenv-DsRed2 (kindly provided by Miguel Quiñones-Mateu) were transferred into pNL4-3-MSS (Multiple Silent Site), using *Bam*HI (position 8465 in NL4-3) and *Xho*I (position 8887 in NL4-3) (New England Biolabs) restriction sites, to generate the infectious molecular clones pNL4-3-MSS-eGFP and pNL4-3-MSS-DsRed2 (**Fig. 1A**). The resulting plasmids, NL4-3-MSS-eGFP and NL4-3-MSS-DsRed2, were sequenced across the junctions 5′ and 3′ of the fluorescent reporter gene using primers overlapping the 5′ *Bam*HI site (5′-TAGTGAACGGATCCTTAGCACTTATC-3′) and 3′ *Xho*I site (5′-TTCTAGGTCTCGAGATACTGCTCCCAC-3′).

Chimeric infectious molecular clones in the Env region were generated by PCR amplifying archival clones of 1157i C2-V4 sequences in pGEM-T with primers containing the *Age*I (5′-CACATGGAATCAG*ACCGGT*AGTATC-3′) and *Sbf*I (5′-GTTTTAT*CCTGCAGG*GGAGTGTGATTG-3′) restriction sites to exactly match the sites flanking C2-V4 in the pNL4-3-MSS vector (position 6970 to 7464 in NL4-3). The resulting PCR products were digested with the appropriate enzymes (New England Biolabs) and ligated into a pSP72 subcloning vector, containing *EcoR*I-*Xho*I sequence from NL4-3-MSS. The chimeric *env* sequences from the pSP72 vector were then transferred into NL4-3-MSS-eGFP and NL4-3-MSS-DsRed2 utilizing the *Eco*RI (position 5743) and *Bam*HI (position 8465) restriction sites, to produce a panel of infectious molecular clones identical to one another, except at the C2-V4 sequence derived from Patient 1157.

### NL4-3-MSS-eGFP, NL4-3-MSS-DsRed2 Nef Protein Expression

In order to make genomic RNA and all of the necessary mRNAs for protein production from a single LTR promoter, retroviruses employ an intricate system of splice donors, splice acceptors, and accessory proteins to regulate expression. Inserting fluorescent proteins 5′ of the *nef* gene could disrupt Nef production. To assess Nef protein expression, a control Nef expression construct pcDNA-Nef was generated by PCR amplifying the NL4-3 *nef* gene (Forward: 5′-CACCATGGGTGGCAAGT-3′, Reverse: 5′-GCAGTTCTTGAAGTACTCCGG-3′) and transferring it into a pcDNA3.1 Directional TOPO vector (Invitrogen). HEK293T cells in 6-well plates were transfected with 2 µg of the following plasmids using Fugene6 according to manufacturer’s instructions (Roche): pNL4-3-Δenv-eGFP [94], pcDNA-Nef, NL4-3-MSS, NL4-3-MSS-eGFP or NL4-3-MSS-DsRed2. 24-hours post transfection, cells were starved for Cys/Met, followed by radiolabeling with [^35^S]-Cys/Met (MP biomedicals) at 100 µCi/mL for one hour. Cells were lysed (1% NP40, 0.1% SDS, 0.5% DOC in sterile PBS) and immunoprecipitated with anti-Nef hyperimmune sera (Advanced BioScience Laboratories, Inc., #5416). Proteins were resolved on a 12% SDS-PAGE and quantified by phosphorimager analysis. This procedure was repeated with mock-transfected cells, NL4-3-MSS, NL4-3-MSS-eGFP, NL4-3-MSS-DsRed2, and with the original pNL4-3-Δenv-eGFP and pNL4-3-Δenv-DsRed2 as described in Weber et al.

### Replication Kinetics

Supernatants from NL4-3-MSS-eGFP and NL4-3-MSS-DsRed2 transfections were 0.45 µm filtered 48-hours post-transfection and assayed in triplicate for reverse transcriptase activity according to manufacturer’s instructions (GE, Quan-T-RT assay system, TRK1022). In a 6-well plate, 10^5^ U87.CD4.CXCR4 were infected with equivalent units of RT activity of NL4-3-MSS- eGFP, NL4-3-MSS-DsRed2, or Mock. Three hours post-infection, cells were washed with PBS, and returned to U87 growth media. Aliquots of 200 µl of infected cell supernatant from days 2, 4, 6, 8, 10, and 12 post-infection were filtered and stored at -80 C until the completion of the time course, at which time 50 µl of each was used to quantify RT activity in triplicate.

### NL4-3-MSS-eGFP and NL4-3-MSS-DsRed2 Mono and Dual Infections

NL4-3-MSS-eGFP and NL4-3-MSS-DsRed2 transfection supernatants were filtered 48-hours post-transfection, and titers of viral stocks were calculated via limiting dilution on TZM-bl indicator cells. In a 96-well plate, 20,000 TZM-bl cells were infected with serial dilutions of viral stocks, in triplicate. Forty-eight hours post-infection, cells were stained *in situ* for *beta*-galactosidase activity. Infectious units (TCID_50_/ml) were calculated according to the Reed and Muench method based on staining endpoints.

Using a MOI of 1.0, 0.1, or 0.01, 20,000 U87.CD4.CXCR4 were infected in triplicate with NL4-3-MSS-eGFP and NL4-3-MSS-DsRed2 in mono- and dual-infections. Five days post-infection, U87 cells were trypsinized, resuspended in PBS, and fixed with paraformaldehyde (final concentration of 0.25%). Fluorescent cells were quantified from counting 10,000 events on an Influx cell sorter, excitation 488-nm (eGFP) and 561-nm (DsRed2). Data was analyzed using Summit v4.3 software.

### Quantification of Chimera Fitness

All Env chimeras utilized CCR5 exclusively as a coreceptor (not shown) as anticipated by the sequences of the V3 terminal tetrapeptide. Env chimera virus stocks were prepared and titered as above. For dual-infection competitions in PBMC, CD4+ T-cells from three donors were aliquoted into 20 wells of a 24-well plate for individual mock, mono-, and dual infections for the five time-points. Cells were infected at an MOI of 10. On days 3-7, fluorescent cells were quantified by counting 50,000 events on an Influx cell sorter, in triplicate, using the 488-nm (eGFP) and 561-nm (DsRed2) laser lines. Data was analyzed using Cytopeia software.

Relative fitness values (W) were calculated as follows [45], [48], [85]:

W_RED_ = 2×(Dual_RED_/Mono_RED_)/(Dual_RED_/Mono_RED_)+(Dual_GFP_/Mono_GFP_).

W_GFP_ = 2×(Dual_GFP_/Mono_GFP_)/(Dual_GFP_/Mono_GFP_)+(Dual_RED_/Mono_RED_).

For dual-infection competitions in U87.CD4.CCR5 cells, all chimeras (12 in total) were competed against all others (DsRed2 versus eGFP) in triplicate, generating 36 data points for each chimera (including self-self competitions, used for normalization). Briefly, in a 96-well plate, 2×10^4^ U87.CD4.CCR5 cells were infected in triplicate at a MOI of 0.1 for eGFP and DsRed2 mono-infections, eGFP/DsRed2 dual infections were conducted at 0.1 MOI of each chimera, and mock infections served as controls. Five days post-infection, the infected and dually infected cultures were analyzed by flow cytometry as above.

### Processing, Incorporation, and Infectivity of Chimeric Envelopes

HEK293T cells were transfected with infant 1157 pNL4-3 eGFP chimeras in quadruplet as described above. Twenty-four hours post-transfection, cells were starved for Cys/Met and pulse-labeled with [^35^S]-Cys/Met (MP Biomedicals) at 100 µCi/mL for one hour. Cells were lysed (1% NP40, 0.1% SDS, 0.5% DOC in sterile filtered PBS) at time-point 0 for the ‘pulse’, and complete media was added to replicate well for 4 hours, 8 hours, and 24 hours ‘chase’ time-points. HIV-1 proteins were immunoprecipitated with HIV-Ig (AIDS Research and Reference Reagent Program, Division of AIDS, NIAID, NIH: Catalog #3957, HIV-IG from NABI and NHLBI) and pelleted with *Staphylococcus aureus* membranes. Lysates were resolved on 9% SDS-PAGE and the labeled proteins were quantified by phosphorimagery (Optiquant software).

Labeled supernatants from the 24-hour time-point were filtered and 1 ml aliquots were pelleted in a Beckman TLA-55 at 136,000×*g* at 4°C for one hour. Viral pellets were lysed as above, and immunoprecipitated with HIV-Ig and *S. aureus* membranes. Viral proteins were resolved by 9% SDS-PAGE. Phosphorimager analysis was used to quantify ratios of gp120 to p66 (RT) as an index of Env incorporation. The remainder of the radiolabeled viral stocks was used to infect 2×10^4^ TZM-bl cells, in triplicate. Infectivity was assayed 24-hours post-infection using HIV-induced luciferase expression (Promega, Victor3). Relative light units produced in each infection was normalized to the amount of p66 (from the IP of the viral pellet) in the preparation (RLU/p66) and this quantity was, in turn, normalized to the incorporation index (Env/p66). Thus this led to quantification of the RLU/Env, or the infectivity/Env.

### Receptor Affinity via Competition with Anti-CD4 or Anti-CCR5 Antibodies

To achieve simultaneous addition of virus and antibody to target cells, 1157 Env chimeric NL4-3 eGFP viral stocks at a MOI of 0.1 were incubated with serial dilutions of anti-CD4 antibody, B4 (NIH AIDS Research and Reference Reagent Program, Division of AIDS, NIAID, NIH: Cell Surface CD4 Complex Monoclonal B4 from United Biomedical Inc. [95]), and the mixture was added to 20,000 TZM-bl cells in triplicate. TZM-bl lysates were analyzed 24 hours post-infection for luciferase expression (Promega) and the IC_50_ concentration of B4 antibody was calculated relative to infections without added B4 antibody. To assess the affinity of the Env CCR5 interaction, the analysis was repeated with anti-CCR5 antibody, 2D7 (NIH AIDS Research and Reference Reagent Program, Division of AIDS, NIAID, NIH: MAb to CCR5 (2D7)). YU2 (a CCR5 tropic virus) and wild-type NL4-3 (a CXCR4 tropic virus) were included as controls in both experiments (**Fig. S5**).

### Statistical Analyses

Analyses were carried out using Prism 5.0c for Mac software. For replication kinetics experiments, the time required to achieve 50% maximal RT activity (RT_50_) and the ‘HillSlope’ of the increase in RT activity was calculated by the ‘log(agonist) vs. response – Variable slope (four parameters)’ subroutine in Prism (Y = Bottom+(Top-Bottom)/(1+10̂((LogEC50-X)*HillSlope)). For statistical analysis of wild type dual-infection competitions, the number of fluorescent events in wild-type mono- and dual infections were compared by 1-way ANOVA at a MOI of 0.1 and 0.01. A one sample *t*-test was used to compare average fitness values of chimeras to a theoretical value of 1.0 (neutrality), with a 99% Confidence Interval, to test for deviations from neutrality. To determine whether average fitness values were different from one another, the value for each chimera was compared to all others via a Bonferroni's Multiple Comparison Test. The IC_50_ of B4 and 2D7 antibodies was calculated utilizing logarithmic regression equations contained within Microsoft Excel 2004 for Mac. Correlations were determined by plotting the relative fitness values versus the parameter of interest, and by calculating the Pearson’s correlation coefficient (R^2^) of the trendline along with 95% confidence intervals (CI).

## Supporting Information

Figure S1 Nef is not expressed from NL4-3-MSS-eGFP or DsRed2, or ancestral constructs. 293T cells were transfected with mock (M), pNL4-3-Δenv-eGFP [94] (ΔE), pcDNA-Nef-V5-6xHis (V5-Nef), NL4-3-MSS (C), NL4-3-MSS-eGFP (G) or NL4-3-MSS-DsRed2 (R) (left panel) and NL4-3-MSS, NL4-3-MSS-eGFP, NL4-3-MSS-DsRed2, and with the original pNL4-3-Δenv-eGFP (ΔEG), and pNL4-3-Δenv-DsRed2 (ΔER) [50]. ^35^S radiolabeled transfections were lysed, immunoprecipitated with Nef hyperimmune sera, and resolved by SDS-PAGE. If Nef is required, expression can be regained by inserting a T2A ribosomal skip sequence at the end of the fluorescent reporter, as described in Edmonds et al. [96].
(TIF)

Figure S2 Increase in nucleotide and amino acid differences over time. Average intra time-point differences in nucleotide (blue) and amino acid (red) sequence over time (N = 23-38 sequences) based on data from Ref. 60. Over the 67-month sampling period, average nucleotide diversity within time-points increased at a rate of 0.25±0.045 differences per month; with average amino acid diversity within time-points increased at a rate of 0.18±0.034 per month. Both were statistically significant positive slopes (p = 0.0028 and 0.0030 relatively).
(TIF)

Figure S3 Phylogenetic and amino acid analysis of infant 1157 *env* C2-V4 sequences. Patient *env* C2-V4 DNA sequences were aligned with Sequencher 4.8. The resulting alignment was used to generate an unrooted tree with PhylML [97]. Amino acid sequences were generated from DNA Strider 1.4. Only positions where amino acids differ between two or more sequences are shown. Variable-3 region is highlighted in yellow.
(TIF)

Figure S4 Results from individual patient chimera competitions. U87.CD4.CCR5 cells were infected at a MOI of 0.1 for all mono- and dual-infections. Five days post-infection, fluorescent events were enumerated by flow cytometry and used to calculate relative fitness values for each competition (see **Fig. 3**). W values for all chimeras from all competitions were plotted individually. Circles represent individual W values, black bars represent means, and error bars represent standard deviation.
(TIF)

Figure S5 NL4-3 and YU2 controls for CD4 and CCR5 affinity assay. NL4-3 (CD4, CXCR4) and YU2 (CD4, CCR5) viral stocks were used as assay controls for the CD4 and CCR5 affinity assays (Fig. 7). Viral stocks were incubated with serial dilutions of a CD4 competitor (B4 Ab) or CCR5 competitor (2D7 Ab), and added to TZB-bl indicator cells. Luciferase activity was quantified and used to calculate IC_50_ concentrations. While both viruses were susceptible to competition with the anti-CD4 Ab B4 (NL4-3 IC_50_ = 4.928 µg/ml), YU2 IC_50_ = 1.075 µg/ml), only YU2 was susceptible to competition with the anti-CCR5 Ab 2D7 (IC_50_ = 0.0280 µg/ml). NL4-3 luciferase activity in the absence of 2D7 (133,516 RLU ±30,279) was similar to luciferase activity at the highest concentrations of 2D7 (0.5 µg/ml) 125,668 RLU ±7,940).
(TIF)

Figure S6 Real-Time reagents directed towards eGFP or DsRed2 are suitable for multiplex reactions. DsRed2 primer-probe set (DsRed2 Forward: 5′-CCTCCTCCGAGAACGTCATC-3′, Reverse: 5′-CCCTCCATGCGCACCTT-3′, Probe: 5′-CCGAGTTCATGCGCTT-3′) was used to detect either 1000 copies of NL4-3-MSS-DsRed2 plasmid (Idealized), 1000 copies of NL4-3-MSS-DsRed2 in the presence of 1000 copies of NL4-3-MSS-eGFP (Template interference), 1000 copies of NL4-3-MSS-DsRed2 in the presence of 1000 copies of NL4-3-MSS-eGFP and the associated eGFP primer-probe set (eGFP Forward: 5′-GGGCACAAGCTGGAGTACAAC-3′, Reverse: 5′-TCTGCTTGTCGGCCATGATA-3′, Probe: 5′-ACAGCCACAACGTCT-3′) (Multiplex), or 10,000 copies of NL4-3-MSS-eGFP (Irrelevant template) (left panel). NL4-3-MSS-eGFP plasmid was detected in a similar manner (right panel). Using Bonferroni’s Multiple Comparison Test, there was no difference between cycle threshold values of Idealized, Template Interference, or Multiplex reactions, indicating that the presence of the alternate fluorescent DNA or reagents does not effect detection. There was a statistically significant difference between those Cts and the irrelevant template (p<0.001), indicating that there is no non-specific amplification of the alternate fluorescent DNA. These primer-probe sets are suitable for multiplex Real-Time PCR.
(TIF)

Table S1 Column statistics of aggregate data. Statistical analysis of Figure 4 data (top panel) and One sample t test comparing average relative fitness values to neutrality (1.0) (bottom panel).
(XLSX)

Table S2 Bonferroni’s Multiple Comparison Test. Data from each chimera (**Fig. 4**) was compared against all other chimeras to determine if the were statistically different. Statistically significant differences are noted with stars (*, p<0.05; **, p<0.01; ***, p<0.001).
(XLSX)

## References

[1] Jetzt AE, Yu H, Klarmann GJ, Ron Y, Preston BD, et al (2000) High rate of recombination throughout the human immunodeficiency virus type 1 genome. J Virol 74: 1234–1240. 10627533 10.1128/jvi.74.3.1234-1240.2000 PMC111457

[2] Mansky LM, Temin HM (1995) Lower in vivo mutation rate of human immunodeficiency virus type 1 than that predicted from the fidelity of purified reverse transcriptase. J Virol 69: 5087–5094. 7541846 10.1128/jvi.69.8.5087-5094.1995 PMC189326

[3] Domingo E, Escarmis C, Sevilla N, Moya A, Elena SF, et al (1996) Basic concepts in RNA virus evolution. Faseb J 10: 859–864. 8666162 10.1096/fasebj.10.8.8666162

[4] McMichael AJ, Phillips RE (1997) Escape of human immunodeficiency virus from immune control. Annu Rev Immunol 15: 271–296. 9143689 10.1146/annurev.immunol.15.1.271

[5] Metzner KJ, Giulieri SG, Knoepfel SA, Rauch P, Burgisser P, et al (2009) Minority quasispecies of drug-resistant HIV-1 that lead to early therapy failure in treatment-naive and -adherent patients. Clin Infect Dis 48: 239–247. 19086910 10.1086/595703

[6] Back NK, Nijhuis M, Keulen W, Boucher CA, Oude Essink BO, et al (1996) Reduced replication of 3TC-resistant HIV-1 variants in primary cells due to a processivity defect of the reverse transcriptase enzyme. EMBO J 15: 4040–4049. 8670908 PMC452124

[7] Bangsberg DR (2008) Preventing HIV antiretroviral resistance through better monitoring of treatment adherence. J Infect Dis 197 Suppl 3S272–278. 18447613 10.1086/533415

[8] Croteau G, Doyon L, Thibeault D, McKercher G, Pilote L, et al (1997) Impaired fitness of human immunodeficiency virus type 1 variants with high-level resistance to protease inhibitors. J Virol 71: 1089–1096. 8995629 10.1128/jvi.71.2.1089-1096.1997 PMC191160

[9] Debyser Z, De Vreese K, Knops-Gerrits PP, Baekelandt V, Bhikhabhai R, et al (1993) Kinetics of different human immunodeficiency virus type 1 reverse transcriptases resistant to human immunodeficiency virus type 1-specific reverse transcriptase inhibitors. Mol Pharmacol 43: 521–526. 7682649

[10] Lockman S, Shapiro RL, Smeaton LM, Wester C, Thior I, et al (2007) Response to antiretroviral therapy after a single, peripartum dose of nevirapine. N Engl J Med 356: 135–147. 17215531 10.1056/NEJMoa062876

[11] Lucas GM (2005) Antiretroviral adherence, drug resistance, viral fitness and HIV disease progression: a tangled web is woven. J Antimicrob Chemother 55: 413–416. 15722389 10.1093/jac/dki042

[12] Nijhuis M, Schuurman R, de Jong D, Erickson J, Gustchina E, et al (1999) Increased fitness of drug resistant HIV-1 protease as a result of acquisition of compensatory mutations during suboptimal therapy. AIDS 13: 2349–2359. 10597776 10.1097/00002030-199912030-00006

[13] Richman D, Shih CK, Lowy I, Rose J, Prodanovich P, et al (1991) Human immunodeficiency virus type 1 mutants resistant to nonnucleoside inhibitors of reverse transcriptase arise in tissue culture. Proc Natl Acad Sci U S A 88: 11241–11245. 1722324 10.1073/pnas.88.24.11241 PMC53110

[14] Saen-oon S, Aruksakunwong O, Wittayanarakul K, Sompornpisut P, Hannongbua S (2007) Insight into analysis of interactions of saquinavir with HIV-1 protease in comparison between the wild-type and G48V and G48V/L90M mutants based on QM and QM/MM calculations. J Mol Graph Model 26: 720–727. 17543558 10.1016/j.jmgm.2007.04.009

[15] Sharma PL, Crumpacker CS (1997) Attenuated replication of human immunodeficiency virus type 1 with a didanosine-selected reverse transcriptase mutation. J Virol 71: 8846–8851. 9343245 10.1128/jvi.71.11.8846-8851.1997 PMC192351

[16] Palella FJ Jr, Delaney KM, Moorman AC, Loveless MO, Fuhrer J, et al (1998) Declining morbidity and mortality among patients with advanced human immunodeficiency virus infection. HIV Outpatient Study Investigators. N Engl J Med 338: 853–860. 9516219 10.1056/NEJM199803263381301

[17] Borrow P, Lewicki H, Hahn BH, Shaw GM, Oldstone MB (1994) Virus-specific CD8+ cytotoxic T-lymphocyte activity associated with control of viremia in primary human immunodeficiency virus type 1 infection. J Virol 68: 6103–6110. 8057491 10.1128/jvi.68.9.6103-6110.1994 PMC237022

[18] Migueles SA, Sabbaghian MS, Shupert WL, Bettinotti MP, Marincola FM, et al (2000) HLA B*5701 is highly associated with restriction of virus replication in a subgroup of HIV-infected long term nonprogressors. Proc Natl Acad Sci U S A 97: 2709–2714. 10694578 10.1073/pnas.050567397 PMC15994

[19] Kiepiela P, Ngumbela K, Thobakgale C, Ramduth D, Honeyborne I, et al (2007) CD8+ T-cell responses to different HIV proteins have discordant associations with viral load. Nat Med 13: 46–53. 17173051 10.1038/nm1520

[20] Wang YE, Li B, Carlson JM, Streeck H, Gladden AD, et al (2009) Protective HLA class I alleles that restrict acute-phase CD8+ T-cell responses are associated with viral escape mutations located in highly conserved regions of human immunodeficiency virus type 1. J Virol 83: 1845–1855. 19036810 10.1128/JVI.01061-08 PMC2643763

[21] Koup RA, Safrit JT, Cao Y, Andrews CA, McLeod G, et al (1994) Temporal association of cellular immune responses with the initial control of viremia in primary human immunodeficiency virus type 1 syndrome. J Virol 68: 4650–4655. 8207839 10.1128/jvi.68.7.4650-4655.1994 PMC236393

[22] Altfeld M, Addo MM, Rosenberg ES, Hecht FM, Lee PK, et al (2003) Influence of HLA-B57 on clinical presentation and viral control during acute HIV-1 infection. AIDS 17: 2581–2591. 14685052 10.1097/00002030-200312050-00005

[23] Draenert R, Le Gall S, Pfafferott KJ, Leslie AJ, Chetty P, et al (2004) Immune selection for altered antigen processing leads to cytotoxic T lymphocyte escape in chronic HIV-1 infection. J Exp Med 199: 905–915. 15067030 10.1084/jem.20031982 PMC2211885

[24] Leslie AJ, Pfafferott KJ, Chetty P, Draenert R, Addo MM, et al (2004) HIV evolution: CTL escape mutation and reversion after transmission. Nat Med 10: 282–289. 14770175 10.1038/nm992

[25] Miura T, Brumme CJ, Brockman MA, Brumme ZL, Pereyra F, et al.. (2009) Hla Associated Viral Mutations Are Common in Human Immunodeficiency Virus Type 1 Elite Controllers. J Virol. 10.1128/JVI.02459-08 PMC2655568 19153230

[26] Fryer HR, Frater J, Duda A, Roberts MG, Phillips RE, et al (2010) Modelling the evolution and spread of HIV immune escape mutants. PLoS Pathog 6: e1001196. 21124991 10.1371/journal.ppat.1001196 PMC2987822

[27] Domingo E, Holland JJ (1997) RNA virus mutations and fitness for survival. Annu Rev Microbiol 51: 151–178. 9343347 10.1146/annurev.micro.51.1.151

[28] Nara PL, Smit L, Dunlop N, Hatch W, Merges M, et al (1990) Emergence of viruses resistant to neutralization by V3-specific antibodies in experimental human immunodeficiency virus type 1 IIIB infection of chimpanzees. J Virol 64: 3779–3791. 2370681 10.1128/jvi.64.8.3779-3791.1990 PMC249673

[29] Goulder PJ, Bunce M, Krausa P, McIntyre K, Crowley S, et al (1996) Novel, cross-restricted, conserved, and immunodominant cytotoxic T lymphocyte epitopes in slow progressors in HIV type 1 infection. AIDS Res Hum Retroviruses 12: 1691–1698. 8959245 10.1089/aid.1996.12.1691

[30] Kaslow RA, Carrington M, Apple R, Park L, Munoz A, et al (1996) Influence of combinations of human major histocompatibility complex genes on the course of HIV-1 infection. Nat Med 2: 405–411. 8597949 10.1038/nm0496-405

[31] Geldmacher C, Currier JR, Herrmann E, Haule A, Kuta E, et al (2007) CD8 T-cell recognition of multiple epitopes within specific Gag regions is associated with maintenance of a low steady-state viremia in human immunodeficiency virus type 1-seropositive patients. J Virol 81: 2440–2448. 17182686 10.1128/JVI.01847-06 PMC1865944

[32] Chopera DR, Mlotshwa M, Woodman Z, Mlisana K, de Assis Rosa D, et al (2011) Virological and immunological factors associated with HIV-1 differential disease progression in HLA-B 58:01-positive individuals. J Virol 85: 7070–7080. 21613398 10.1128/JVI.02543-10 PMC3126593

[33] Ogg GS, Jin X, Bonhoeffer S, Dunbar PR, Nowak MA, et al (1998) Quantitation of HIV-1-specific cytotoxic T lymphocytes and plasma load of viral RNA. Science 279: 2103–2106. 9516110 10.1126/science.279.5359.2103

[34] Richman DD, Wrin T, Little SJ, Petropoulos CJ (2003) Rapid evolution of the neutralizing antibody response to HIV type 1 infection. Proc Natl Acad Sci U S A 100: 4144–4149. 12644702 10.1073/pnas.0630530100 PMC153062

[35] Gray ES, Moore PL, Choge IA, Decker JM, Bibollet-Ruche F, et al (2007) Neutralizing antibody responses in acute human immunodeficiency virus type 1 subtype C infection. J Virol 81: 6187–6196. 17409164 10.1128/JVI.00239-07 PMC1900112

[36] Li B, Decker JM, Johnson RW, Bibollet-Ruche F, Wei X, et al (2006) Evidence for potent autologous neutralizing antibody titers and compact envelopes in early infection with subtype C human immunodeficiency virus type 1. J Virol 80: 5211–5218. 16699001 10.1128/JVI.00201-06 PMC1472127

[37] Wei X, Decker JM, Wang S, Hui H, Kappes JC, et al (2003) Antibody neutralization and escape by HIV-1. Nature 422: 307–312. 12646921 10.1038/nature01470

[38] Starcich BR, Hahn BH, Shaw GM, McNeely PD, Modrow S, et al (1986) Identification and characterization of conserved and variable regions in the envelope gene of HTLV-III/LAV, the retrovirus of AIDS. Cell 45: 637–648. 2423250 10.1016/0092-8674(86)90778-6

[39] Albert J, Abrahamsson B, Nagy K, Aurelius E, Gaines H, et al (1990) Rapid development of isolate-specific neutralizing antibodies after primary HIV-1 infection and consequent emergence of virus variants which resist neutralization by autologous sera. AIDS 4: 107–112. 2328092 10.1097/00002030-199002000-00002

[40] Arendrup M, Nielsen C, Hansen JE, Pedersen C, Mathiesen L, et al (1992) Autologous HIV-1 neutralizing antibodies: emergence of neutralization-resistant escape virus and subsequent development of escape virus neutralizing antibodies. J Acquir Immune Defic Syndr 5: 303–307. 1740756

[41] Wilke CO, Wang JL, Ofria C, Lenski RE, Adami C (2001) Evolution of digital organisms at high mutation rates leads to survival of the flattest. Nature 412: 331–333. 11460163 10.1038/35085569

[42] Sardanyes J, Elena SF, Sole RV (2008) Simple quasispecies models for the survival-of-the-flattest effect: The role of space. J Theor Biol 250: 560–568. 18054366 10.1016/j.jtbi.2007.10.027

[43] Marozsan AJ, Moore DM, Lobritz MA, Fraundorf E, Abraha A, et al (2005) Differences in the fitness of two diverse wild-type human immunodeficiency virus type 1 isolates are related to the efficiency of cell binding and entry. J Virol 79: 7121–7134. 15890952 10.1128/JVI.79.11.7121-7134.2005 PMC1112120

[44] Rangel HR, Weber J, Chakraborty B, Gutierrez A, Marotta ML, et al (2003) Role of the human immunodeficiency virus type 1 envelope gene in viral fitness. J Virol 77: 9069–9073. 12885922 10.1128/JVI.77.16.9069-9073.2003 PMC167250

[45] Quinones-Mateu ME, Ball SC, Marozsan AJ, Torre VS, Albright JL, et al (2000) A dual infection/competition assay shows a correlation between ex vivo human immunodeficiency virus type 1 fitness and disease progression. J Virol 74: 9222–9233. 10982369 10.1128/jvi.74.19.9222-9233.2000 PMC102121

[46] Ball SC, Abraha A, Collins KR, Marozsan AJ, Baird H, et al (2003) Comparing the ex vivo fitness of CCR5-tropic human immunodeficiency virus type 1 isolates of subtypes B and C. J Virol. 77: 1021–1038. 10.1128/JVI.77.2.1021-1038.2003 PMC140829 12502818

[47] Kong X, West JT, Zhang H, Shea DM, M’Soka TJ, et al (2008) The human immunodeficiency virus type 1 envelope confers higher rates of replicative fitness to perinatally transmitted viruses than to nontransmitted viruses. J Virol 82: 11609–11618. 18786994 10.1128/JVI.00952-08 PMC2583653

[48] Troyer RM, Collins KR, Abraha A, Fraundorf E, Moore DM, et al (2005) Changes in human immunodeficiency virus type 1 fitness and genetic diversity during disease progression. J Virol 79: 9006–9018. 15994794 10.1128/JVI.79.14.9006-9018.2005 PMC1168764

[49] Holland JJ, de la Torre JC, Clarke DK, Duarte E (1991) Quantitation of relative fitness and great adaptability of clonal populations of RNA viruses. J Virol 65: 2960–2967. 2033662 10.1128/jvi.65.6.2960-2967.1991 PMC240937

[50] Weber J, Weberova J, Carobene M, Mirza M, Martinez-Picado J, et al (2006) Use of a novel assay based on intact recombinant viruses expressing green (EGFP) or red (DsRed2) fluorescent proteins to examine the contribution of pol and env genes to overall HIV-1 replicative fitness. J Virol Methods 136: 102–117. 16690137 10.1016/j.jviromet.2006.04.004

[51] Hwang SS, Boyle TJ, Lyerly HK, Cullen BR (1991) Identification of the envelope V3 loop as the primary determinant of cell tropism in HIV-1. Science 253: 71–74. 1905842 10.1126/science.1905842

[52] Clevestig P, Pramanik L, Leitner T, Ehrnst A (2006) CCR5 use by human immunodeficiency virus type 1 is associated closely with the gp120 V3 loop N-linked glycosylation site. J Gen Virol 87: 607–612. 16476981 10.1099/vir.0.81510-0

[53] Bosch ML, Earl PL, Fargnoli K, Picciafuoco S, Giombini F, et al (1989) Identification of the fusion peptide of primate immunodeficiency viruses. Science 244: 694–697. 2541505 10.1126/science.2541505

[54] Jones PL, Korte T, Blumenthal R (1998) Conformational changes in cell surface HIV-1 envelope glycoproteins are triggered by cooperation between cell surface CD4 and co-receptors. J Biol Chem 273: 404–409. 9417096 10.1074/jbc.273.1.404

[55] Margolis L, Shattock R (2006) Selective transmission of CCR5-utilizing HIV-1: the ‘gatekeeper’ problem resolved? Nat Rev Microbiol 4: 312–317. 16541138 10.1038/nrmicro1387

[56] Lobritz MA, Marozsan AJ, Troyer RM, Arts EJ (2007) Natural variation in the V3 crown of human immunodeficiency virus type 1 affects replicative fitness and entry inhibitor sensitivity. J Virol 81: 8258–8269. 17522224 10.1128/JVI.02739-06 PMC1951322

[57] McKeating JA, Gow J, Goudsmit J, Pearl LH, Mulder C, et al (1989) Characterization of HIV-1 neutralization escape mutants. Aids 3: 777–784. 2483618 10.1097/00002030-198912000-00001

[58] Derdeyn CA, Decker JM, Sfakianos JN, Wu X, O’Brien WA, et al (2000) Sensitivity of human immunodeficiency virus type 1 to the fusion inhibitor T-20 is modulated by coreceptor specificity defined by the V3 loop of gp120. J Virol 74: 8358–8367. 10954535 10.1128/jvi.74.18.8358-8367.2000 PMC116346

[59] Kuhmann SE, Pugach P, Kunstman KJ, Taylor J, Stanfield RL, et al (2004) Genetic and phenotypic analyses of human immunodeficiency virus type 1 escape from a small-molecule CCR5 inhibitor. J Virol 78: 2790–2807. 14990699 10.1128/JVI.78.6.2790-2807.2004 PMC353740

[60] Zhang H, Hoffmann F, He J, He X, Kankasa C, et al (2005) Evolution of subtype C HIV-1 Env in a slowly progressing Zambian infant. Retrovirology 2: 67. 16274482 10.1186/1742-4690-2-67 PMC1308862

[61] Abraha A, Troyer RM, Quinones-Mateu ME, Arts EJ (2005) Methods to determine HIV-1 ex vivo fitness. Methods Mol Biol 304: 355–368. 16061989 10.1385/1-59259-907-9:355

[62] Tso FY, Tully DC, Gonzalez S, Quince C, Ho O, et al (2012) Dynamics of envelope evolution in clade C SHIV-infected pig-tailed macaques during disease progression analyzed by ultra-deep pyrosequencing. PLoS One 7: e32827. 22427893 10.1371/journal.pone.0032827 PMC3299704

[63] Tso FY, Hoffmann FG, Tully DC, Lemey P, Rasmussen RA, et al (2009) A comparative study of HIV-1 clade C env evolution in a Zambian infant with an infected rhesus macaque during disease progression. AIDS 23: 1817–1828. 19609201 10.1097/QAD.0b013e32832f3da6 PMC2901162

[64] Tebit DM, Nankya I, Arts EJ, Gao Y (2007) HIV diversity, recombination and disease progression: how does fitness “fit” into the puzzle? AIDS Rev 9: 75–87. 17694675

[65] Anderson RM, May RM (1996) The population biology of the interaction between HIV-1 and HIV-2: coexistence or competitive exclusion? AIDS 10: 1663–1673. 8970687 10.1097/00002030-199612000-00011

[66] Arien KK, Abraha A, Quinones-Mateu ME, Kestens L, Vanham G, et al (2005) The replicative fitness of primary human immunodeficiency virus type 1 (HIV-1) group M, HIV-1 group O, and HIV-2 isolates. J Virol 79: 8979–8990. 15994792 10.1128/JVI.79.14.8979-8990.2005 PMC1168791

[67] Biesinger T, Kimata JT (2008) HIV-1 Transmission, Replication Fitness and Disease Progression. Virology (Auckl) 2008: 49–63. 20354593 PMC2846839

[68] Keele BF, Giorgi EE, Salazar-Gonzalez JF, Decker JM, Pham KT, et al (2008) Identification and characterization of transmitted and early founder virus envelopes in primary HIV-1 infection. Proc Natl Acad Sci U S A 105: 7552–7557. 18490657 10.1073/pnas.0802203105 PMC2387184

[69] Kimata JT (2006) HIV-1 fitness and disease progression: insights from the SIV-macaque model. Curr HIV Res 4: 65–77. 16454712 10.2174/157016206775197628

[70] Gause GF (1934) Experimental Analysis of Vito Volterra’s Mathematical Theory of the Struggle for Existence. Science 79: 16–17. 10.1126/science.79.2036.16-a 17821472

[71] Clarke DK, Duarte EA, Elena SF, Moya A, Domingo E, et al (1994) The red queen reigns in the kingdom of RNA viruses. Proc Natl Acad Sci U S A 91: 4821–4824. 8197141 10.1073/pnas.91.11.4821 PMC43880

[72] Sole RV, Ferrer R, Gonzalez-Garcia I, Quer J, Domingo E (1999) Red queen dynamics, competition and critical points in a model of RNA virus quasispecies. J Theor Biol 198: 47–59. 10329114 10.1006/jtbi.1999.0901

[73] Weber J, Rangel HR, Chakraborty B, Tadele M, Martinez MA, et al (2003) A novel TaqMan real-time PCR assay to estimate ex vivo human immunodeficiency virus type 1 fitness in the era of multi-target (pol and env) antiretroviral therapy. J Gen Virol 84: 2217–2228. 12867654 10.1099/vir.0.19123-0

[74] Anastassopoulou CG, Marozsan AJ, Matet A, Snyder AD, Arts EJ, et al (2007) Escape of HIV-1 from a small molecule CCR5 inhibitor is not associated with a fitness loss. PLoS Pathog 3: e79. 17542646 10.1371/journal.ppat.0030079 PMC1885273

[75] Song H, Pavlicek JW, Cai F, Bhattacharya T, Li H, et al (2012) Impact of immune escape mutations on HIV-1 fitness in the context of the cognate transmitted/founder genome. Retrovirology 9: 89. 23110705 10.1186/1742-4690-9-89 PMC3496648

[76] Schlub TE, Smyth RP, Grimm AJ, Mak J, Davenport MP (2010) Accurately measuring recombination between closely related HIV-1 genomes. PLoS Comput Biol 6: e1000766. 20442872 10.1371/journal.pcbi.1000766 PMC2861704

[77] Batorsky R, Kearney MF, Palmer SE, Maldarelli F, Rouzine IM, et al (2011) Estimate of effective recombination rate and average selection coefficient for HIV in chronic infection. Proc Natl Acad Sci U S A 108: 5661–5666. 21436045 10.1073/pnas.1102036108 PMC3078368

[78] Chalfie M, Tu Y, Euskirchen G, Ward WW, Prasher DC (1994) Green fluorescent protein as a marker for gene expression. Science 263: 802–805. 8303295 10.1126/science.8303295

[79] Yanushevich YG, Staroverov DB, Savitsky AP, Fradkov AF, Gurskaya NG, et al (2002) A strategy for the generation of non-aggregating mutants of Anthozoa fluorescent proteins. FEBS Lett 511: 11–14. 11821040 10.1016/s0014-5793(01)03263-x

[80] Zhang H, Hoffmann F, He J, He X, Kankasa C, et al (2006) Characterization of HIV-1 subtype C envelope glycoproteins from perinatally infected children with different courses of disease. Retrovirology 3: 73. 17054795 10.1186/1742-4690-3-73 PMC1635063

[81] Javaherian K, Langlois AJ, LaRosa GJ, Profy AT, Bolognesi DP, et al (1990) Broadly neutralizing antibodies elicited by the hypervariable neutralizing determinant of HIV-1. Science 250: 1590–1593. 1703322 10.1126/science.1703322

[82] Corzana F, Busto JH, Jimenez-Oses G, Garcia de Luis M, Asensio JL, et al (2007) Serine versus threonine glycosylation: the methyl group causes a drastic alteration on the carbohydrate orientation and on the surrounding water shell. J Am Chem Soc 129: 9458–9467. 17616194 10.1021/ja072181b

[83] Bunnik EM, Lobbrecht MS, van Nuenen AC, Schuitemaker H (2010) Escape from autologous humoral immunity of HIV-1 is not associated with a decrease in replicative capacity. Virology 397: 224–230. 19945135 10.1016/j.virol.2009.11.009

[84] van Gils MJ, Bunnik EM, Burger JA, Jacob Y, Schweighardt B, et al (2010) Rapid escape from preserved cross-reactive neutralizing humoral immunity without loss of viral fitness in HIV-1-infected progressors and long-term nonprogressors. J Virol 84: 3576–3585. 20071586 10.1128/JVI.02622-09 PMC2838121

[85] Troyer RM, McNevin J, Liu Y, Zhang SC, Krizan RW, et al (2009) Variable fitness impact of HIV-1 escape mutations to cytotoxic T lymphocyte (CTL) response. PLoS Pathog 5: e1000365. 19343217 10.1371/journal.ppat.1000365 PMC2659432

[86] Sather DN, Carbonetti S, Kehayia J, Kraft Z, Mikell I, et al (2012) Broadly neutralizing antibodies developed by an HIV-positive elite neutralizer exact a replication fitness cost on the contemporaneous virus. J Virol 86: 12676–12685. 22973035 10.1128/JVI.01893-12 PMC3497623

[87] Bar KJ, Tsao CY, Iyer SS, Decker JM, Yang Y, et al (2012) Early low-titer neutralizing antibodies impede HIV-1 replication and select for virus escape. PLoS Pathog 8: e1002721. 22693447 10.1371/journal.ppat.1002721 PMC3364956

[88] Mikell I, Sather DN, Kalams SA, Altfeld M, Alter G, et al (2011) Characteristics of the earliest cross-neutralizing antibody response to HIV-1. PLoS Pathog 7: e1001251. 21249232 10.1371/journal.ppat.1001251 PMC3020924

[89] Lassen KG, Lobritz MA, Bailey JR, Johnston S, Nguyen S, et al (2009) Elite suppressor-derived HIV-1 envelope glycoproteins exhibit reduced entry efficiency and kinetics. PLoS Pathog 5: e1000377. 19360131 10.1371/journal.ppat.1000377 PMC2661022

[90] Sterjovski J, Churchill MJ, Roche M, Ellett A, Farrugia W, et al (2011) CD4-binding site alterations in CCR5-using HIV-1 envelopes influencing gp120-CD4 interactions and fusogenicity. Virology 410: 418–428. 21216423 10.1016/j.virol.2010.12.010

[91] Thenin S, Samleerat T, Tavernier E, Ngo-Giang-Huong N, Jourdain G, et al (2012) Envelope glycoproteins of human immunodeficiency virus type 1 variants issued from mother-infant pairs display a wide spectrum of biological properties. Virology 426: 12–21. 22310702 10.1016/j.virol.2012.01.017

[92] Platt EJ, Wehrly K, Kuhmann SE, Chesebro B, Kabat D (1998) Effects of CCR5 and CD4 cell surface concentrations on infections by macrophagetropic isolates of human immunodeficiency virus type 1. J Virol 72: 2855–2864. 9525605 10.1128/jvi.72.4.2855-2864.1998 PMC109730

[93] Bjorndal A, Deng H, Jansson M, Fiore JR, Colognesi C, et al (1997) Coreceptor usage of primary human immunodeficiency virus type 1 isolates varies according to biological phenotype. J Virol 71: 7478–7487. 9311827 10.1128/jvi.71.10.7478-7487.1997 PMC192094

[94] Pierson TC, Zhou Y, Kieffer TL, Ruff CT, Buck C, et al (2002) Molecular characterization of preintegration latency in human immunodeficiency virus type 1 infection. J Virol 76: 8518–8531. 12163571 10.1128/JVI.76.17.8518-8531.2002 PMC136977

[95] Wang CY, Sawyer LS, Murthy KK, Fang X, Walfield AM, et al (1999) Postexposure immunoprophylaxis of primary isolates by an antibody to HIV receptor complex. Proc Natl Acad Sci U S A 96: 10367–10372. 10468614 10.1073/pnas.96.18.10367 PMC17894

[96] Edmonds TG, Ding H, Yuan X, Wei Q, Smith KS, et al (2010) Replication competent molecular clones of HIV-1 expressing Renilla luciferase facilitate the analysis of antibody inhibition in PBMC. Virology 408: 1–13. 20863545 10.1016/j.virol.2010.08.028 PMC2993081

[97] Guindon S, Gascuel O (2003) A simple, fast, and accurate algorithm to estimate large phylogenies by maximum likelihood. Syst Biol 52: 696–704. 14530136 10.1080/10635150390235520

