# Supplementary figures and images for: HIV-1 Env C2-V4 Diversification in a Slow-Progressor Infant Reveals a Flat but Rugged Fitness Landscape
Source: PLoS One. 2013 Apr 29;8(4):e63094. doi: 10.1371/journal.pone.0063094 (PMC3639246; doi:10.1371/journal.pone.0063094)

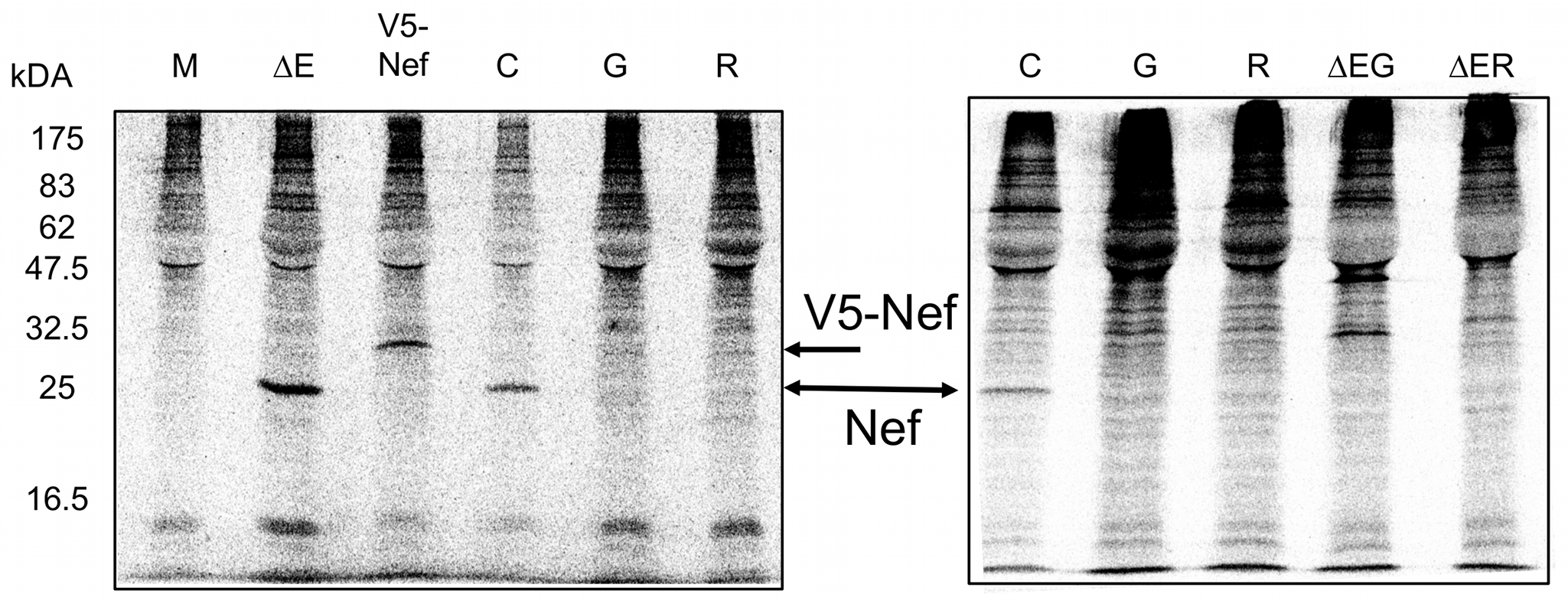

Supplement: Figure S1 — Nef is not expressed from NL4-3-MSS-eGFP or DsRed2, or ancestral constructs. 293T cells were transfected with mock (M), pNL4-3-Δenv-eGFP [94] (ΔE), pcDNA-Nef-V5-6xHis (V5-Nef), NL4-3-MSS (C), NL4-3-MSS-eGFP (G) or NL4-3-MSS-DsRed2 (R) (left panel) and NL4-3-MSS, NL4-3-MSS-eGFP, NL4-3-MSS-DsRed2, and with the original pNL4-3-Δenv-eGFP (ΔEG), and pNL4-3-Δenv-DsRed2 (ΔER) [50]. 35S radiolabeled transfections were lysed, immunoprecipitated with Nef hyperimmune sera, and resolved by SDS-PAGE. If Nef is required, expression can be regained by inserting a T2A ribosomal skip sequence at the end of the fluorescent reporter, as described in Edmonds et al. [96]. (TIF) [file pone.0063094.s001.tif]

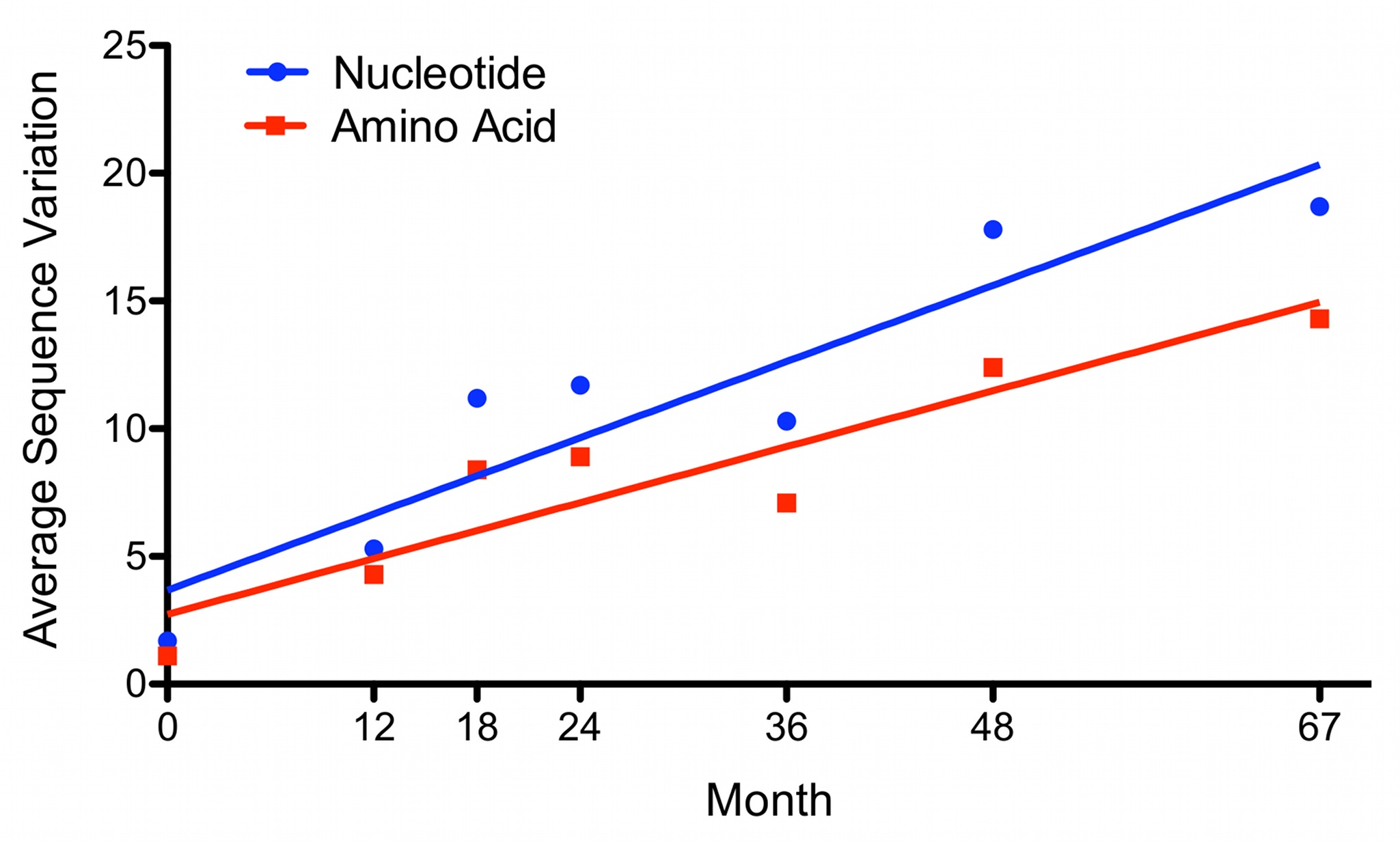

Supplement: Figure S2 — Increase in nucleotide and amino acid differences over time. Average intra time-point differences in nucleotide (blue) and amino acid (red) sequence over time (N = 23-38 sequences) based on data from Ref. 60. Over the 67-month sampling period, average nucleotide diversity within time-points increased at a rate of 0.25±0.045 differences per month; with average amino acid diversity within time-points increased at a rate of 0.18±0.034 per month. Both were statistically significant positive slopes (p = 0.0028 and 0.0030 relatively). (TIF) [file pone.0063094.s002.tif]

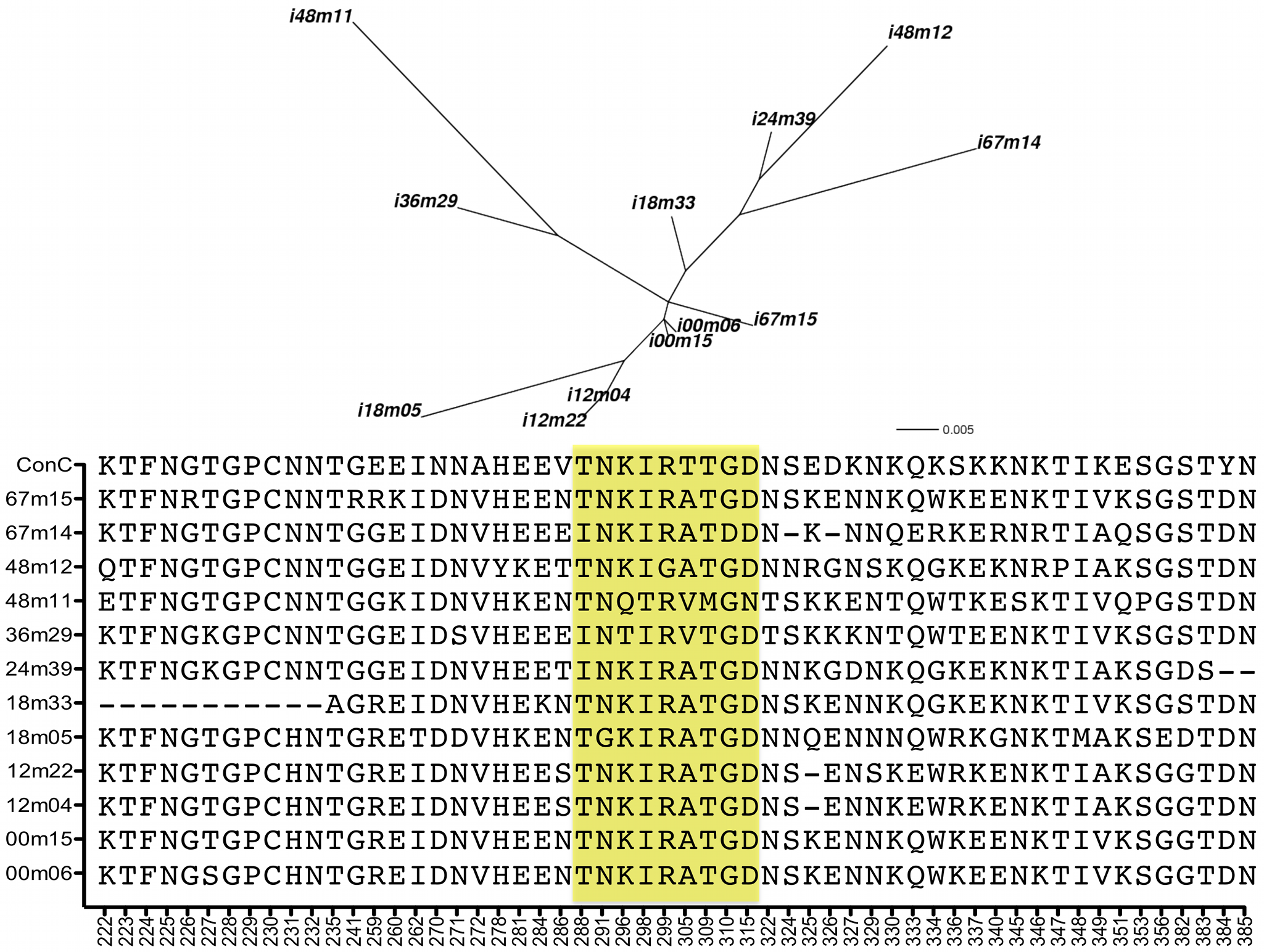

Supplement: Figure S3 — Phylogenetic and amino acid analysis of infant 1157 env C2-V4 sequences. Patient env C2-V4 DNA sequences were aligned with Sequencher 4.8. The resulting alignment was used to generate an unrooted tree with PhylML [97]. Amino acid sequences were generated from DNA Strider 1.4. Only positions where amino acids differ between two or more sequences are shown. Variable-3 region is highlighted in yellow. (TIF) [file pone.0063094.s003.tif]

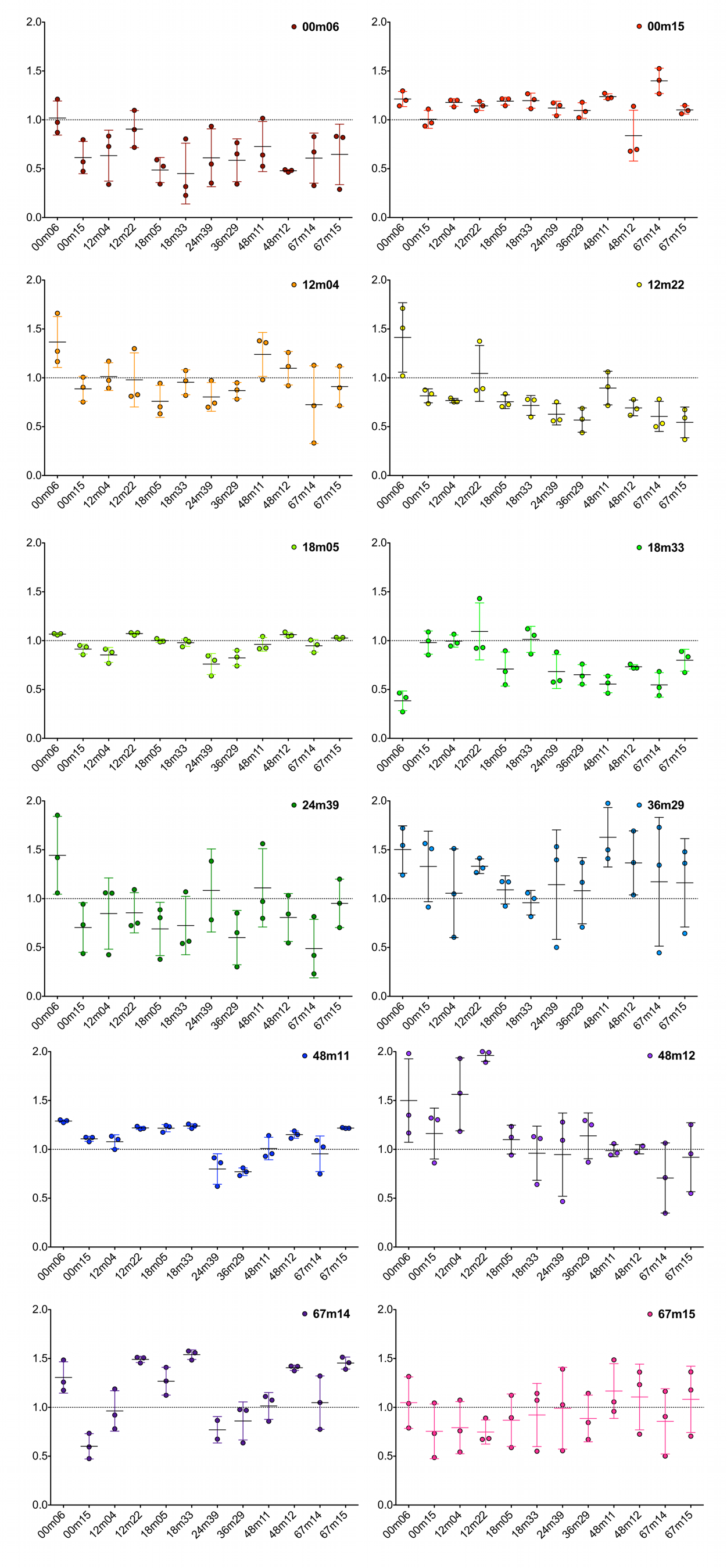

Supplement: Figure S4 — Results from individual patient chimera competitions. U87.CD4.CCR5 cells were infected at a MOI of 0.1 for all mono- and dual-infections. Five days post-infection, fluorescent events were enumerated by flow cytometry and used to calculate relative fitness values for each competition (see Fig. 3 ). W values for all chimeras from all competitions were plotted individually. Circles represent individual W values, black bars represent means, and error bars represent standard deviation. (TIF) [file pone.0063094.s004.tif]

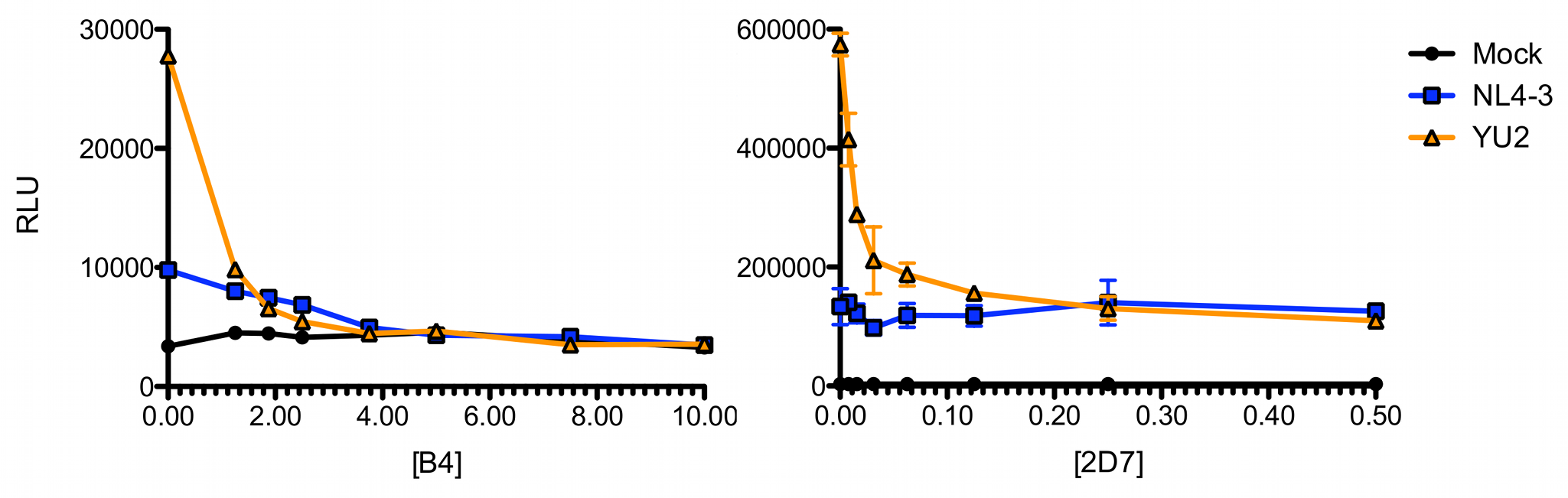

Supplement: Figure S5 — NL4-3 and YU2 controls for CD4 and CCR5 affinity assay. NL4-3 (CD4, CXCR4) and YU2 (CD4, CCR5) viral stocks were used as assay controls for the CD4 and CCR5 affinity assays (Fig. 7). Viral stocks were incubated with serial dilutions of a CD4 competitor (B4 Ab) or CCR5 competitor (2D7 Ab), and added to TZB-bl indicator cells. Luciferase activity was quantified and used to calculate IC50 concentrations. While both viruses were susceptible to competition with the anti-CD4 Ab B4 (NL4-3 IC50 = 4.928 µg/ml), YU2 IC50 = 1.075 µg/ml), only YU2 was susceptible to competition with the anti-CCR5 Ab 2D7 (IC50 = 0.0280 µg/ml). NL4-3 luciferase activity in the absence of 2D7 (133,516 RLU ±30,279) was similar to luciferase activity at the highest concentrations of 2D7 (0.5 µg/ml) 125,668 RLU ±7,940). (TIF) [file pone.0063094.s005.tif]

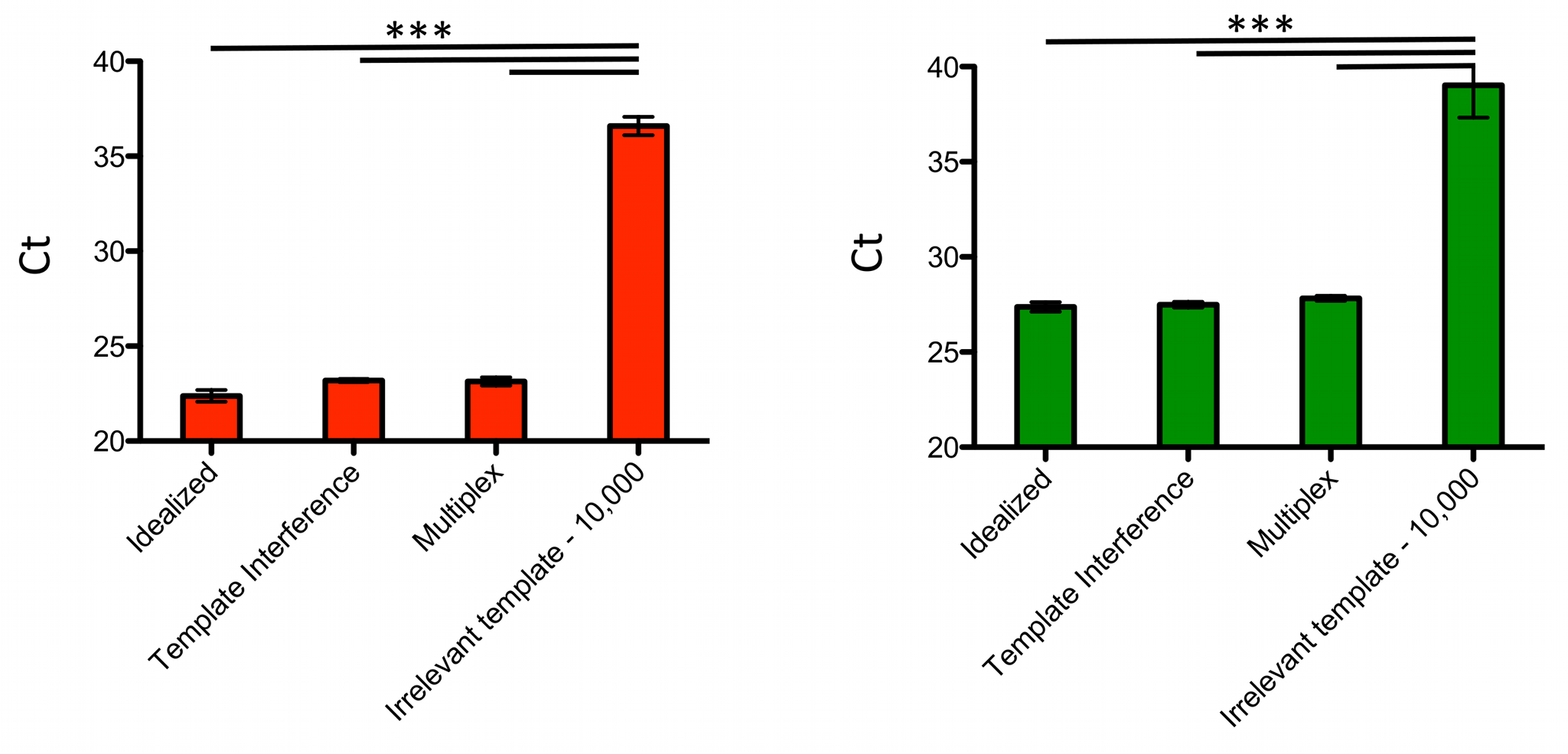

Supplement: Figure S6 — Real-Time reagents directed towards eGFP or DsRed2 are suitable for multiplex reactions. DsRed2 primer-probe set (DsRed2 Forward: 5′-CCTCCTCCGAGAACGTCATC-3′, Reverse: 5′-CCCTCCATGCGCACCTT-3′, Probe: 5′-CCGAGTTCATGCGCTT-3′) was used to detect either 1000 copies of NL4-3-MSS-DsRed2 plasmid (Idealized), 1000 copies of NL4-3-MSS-DsRed2 in the presence of 1000 copies of NL4-3-MSS-eGFP (Template interference), 1000 copies of NL4-3-MSS-DsRed2 in the presence of 1000 copies of NL4-3-MSS-eGFP and the associated eGFP primer-probe set (eGFP Forward: 5′-GGGCACAAGCTGGAGTACAAC-3′, Reverse: 5′-TCTGCTTGTCGGCCATGATA-3′, Probe: 5′-ACAGCCACAACGTCT-3′) (Multiplex), or 10,000 copies of NL4-3-MSS-eGFP (Irrelevant template) (left panel). NL4-3-MSS-eGFP plasmid was detected in a similar manner (right panel). Using Bonferroni’s Multiple Comparison Test, there was no difference between cycle threshold values of Idealized, Template Interference, or Multiplex reactions, indicating that the presence of the alternate fluorescent DNA or reagents does not effect detection. There was a statistically significant difference between those Cts and the irrelevant template (p<0.001), indicating that there is no non-specific amplification of the alternate fluorescent DNA. These primer-probe sets are suitable for multiplex Real-Time PCR. (TIF) [file pone.0063094.s006.tif]
